# Disorders of representation and control in semantic cognition: Effects of familiarity, typicality, and specificity

**DOI:** 10.1016/j.neuropsychologia.2015.04.015

**Published:** 2015-09

**Authors:** Timothy T. Rogers, Karalyn Patterson, Elizabeth Jefferies, Matthew A. Lambon Ralph

**Affiliations:** a MRC Cognition & Brain Sciences Unit, Cambridge, UK; b Department of Psychology, University of Wisconsin-Madison, USA; c Department of Clinical Neurosciences, University of Cambridge, UK; d Department of Psychology, University of York, UK; e Neuroscience and Aphasia Research Unit, School of Psychological Sciences, University of Manchester, UK

## Abstract

We present a case-series comparison of patients with cross-modal semantic impairments consequent on either (a) bilateral anterior temporal lobe atrophy in semantic dementia (SD) or (b) left-hemisphere fronto-parietal and/or posterior temporal stroke in semantic aphasia (SA). Both groups were assessed on a new test battery designed to measure how performance is influenced by concept familiarity, typicality and specificity. In line with previous findings, performance in SD was strongly modulated by all of these factors, with better performance for more familiar items (regardless of typicality), for more typical items (regardless of familiarity) and for tasks that did not require very specific classification, consistent with the gradual degradation of conceptual knowledge in SD. The SA group showed significant impairments on all tasks but their sensitivity to familiarity, typicality and specificity was more variable and governed by task-specific effects of these factors on controlled semantic processing. The results are discussed with reference to theories about the complementary roles of representation and manipulation of semantic knowledge.

## Highlights

• Case-series comparison of semantic dementia (SD) and semantic aphasia (SA).
• Effects of concept familiarity, typicality and specificity across four tasks.
• SD performance was better for familiar/typical items and less specific classification.
• In SA, effects of these factors varied across tasks reflecting the control demands.
• Data are consistent with interacting semantic representations and control processes.

## 1 Introduction

The ease with which we call to mind information about the items encountered in daily life varies with several factors laid bare by cognitive psychology over the last few decades. The speed and accuracy with which an item is named or categorized, or its attributes inferred or verified, can depend upon properties of the item, such as its overall familiarity (Smith, 1967) and its prototypicality (Rosch et al., 1976b), and upon demands of the task, such as the specificity which the item must be classified (Rosch et al., 1976a), the “prepotency” or automaticity of the response to be generated (Shiffrin and Schneider, 1977), and the degree to which the respondent must adjudicate amongst many potentially correct responses (Thompson-Schill et al., 1999). Such phenomena have long been a focus of study in healthy cognition because they shed light on the representations and processes that underlie human conceptual knowledge, and a variety of different models have been proposed to explain these different effects in healthy adults (Ashby and Maddox, 1993; Logan, 1980; Nosofsky, 1986; Shiffrin and Steyvers, 1997).

Neuropsychological studies of such effects also provide compelling evidence toward the development of semantic models, especially when they document patterns that cannot be anticipated from healthy behavior alone. For example, where healthy adults are faster and more accurate to categorize visually-presented items at the basic level (e.g. “bird”) than at a more general level (e.g. “animal”), patients with semantic dementia (SD)—a progressive dementing illness that gradually erodes semantic knowledge—show the reverse pattern (Rogers and Patterson, 2007). As a contrastive example, patients with SD, like healthy controls, are better at naming high-familiarity than low-familiarity items (Warrington, 1975; Woollams et al., 2008), but patients with cross modal semantic impairments following left-hemisphere stroke can show either no familiarity effect or greatly reduced effects in the same tasks (Jefferies and Lambon Ralph, 2006). Such phenomena demonstrate that impaired semantic abilities are not simply slower and less accurate mirrors of healthy abilities. Instead the factors that influence healthy cognition can exert qualitatively different effects in different varieties of disordered behavior. The contrasting patterns provide evidence important for constraining theories about the representations and processes that support healthy semantic cognition.

Neuropsychological work in this vein has mainly focused on one or two factors taken individually within a particular patient group. Only a handful of studies have directly compared effects across different patient groups (e.g., Corbett et al., 2009b; Jefferies and Lambon Ralph, 2006; Libon et al., 2013; Ogar et al., 2011), and to our knowledge, no study has simultaneously studied the joint influence of multiple factors across multiple tasks in different groups. Yet there are reasons for pursuing such large-scale multi-faceted investigation. First, the interesting factors are often confounded in natural concepts. Items judged to be atypical of their category, for instance, also tend to be less familiar, so it is difficult to know how these factors individually contribute to impaired behavior (McRae et al., 2005). Second, the different factors interact with one another even in healthy cognition. For instance, while typical items are classified more rapidly at the basic level than at more specific levels, the reverse is true for atypical items (Jolicoeur et al., 1984); and while people are faster to classify more familiar items when typicality is controlled, they can be faster to classify unfamiliar but typical items than highly familiar but atypical items (Whittlesea, 2002). To understand how the different factors influence impaired semantic cognition, they must be under simultaneous control. Third, the comparison of such effects across patient groups is necessary to understand whether the factors of interest exert the same influences under any form of semantic impairment, or whether different effects are observed for different semantic syndromes. To the extent that differences across syndromes are observed, these may provide clues about the nature of the deficits in the associated syndromes and about the operation of the healthy system.

The current paper provides the first case-series comparison of the effects of familiarity, typicality and specificity in two different semantic syndromes, across four commonly-used tasks that also vary in other respects relevant to understanding semantic impairment. The patient groups differ in their aetiology and lesion sites, and their impairments are posited to stem from disruption to fundamentally different underlying cognitive processes (Jefferies and Lambon Ralph, 2006). Thus a central aim of the work, in addition to simply characterizing how familiarity, typicality, and specificity influence disordered behavior across syndromes and tasks, is to assess whether the hypothesized differences between groups can help to explain when and why the two groups exhibit similar or different patterns of behavior. In the remainder of this section, therefore, we briefly describe the patient groups and working hypotheses about the nature of each disorder developed in prior work.

### 1.1 Semantic dementia, semantic aphasia and the controlled semantic cognition (CSC) framework

Our analyses focus on two varieties of semantic impairment stemming from different aetiologies. The first is semantic dementia (SD), a neurodegenerative disorder also known as the temporal-lobe variant of fronto-temporal dementia (Hodges et al., 1992; Snowden et al., 1989). SD is invariably associated with atrophy and hypometabolism centered on the ventral region of the anterior temporal lobes, bilaterally (Acosta-Cabronero et al., 2011). The semantic impairment in SD affects knowledge of all kinds of concepts, across all modalities of testing, and is relatively pure: other aspects of cognition, including perception and attention, episodic and working memory, executive function, reasoning and problem solving, and grammatical and phonological aspects of language, remain normal or near normal until late in the disease (Hodges and Patterson, 2007; Snowden et al., 2001). Despite its global nature, the deterioration of conceptual knowledge observed in SD is structured in three respects: (i) patients perform better for more frequent or familiar items than for less frequent/familiar items (e.g., Funnell, 1995; Lambon Ralph et al., 1998; Warrington, 1975); (ii) patients often retain at least modest knowledge of more prototypical items (e.g., horse) and properties (e.g., a camel has a mouth) in contrast to very degraded information about less prototypical items (e.g., buffalo) and properties (e.g., a camel has a hump) (Rogers et al., 2004b; Woollams et al., 2008); and (iii) more specific names and concepts (e.g., a particular bird is a “robin”) are more vulnerable than more general names and concepts (e.g., the same object is also a “bird” or an “animal”) (Rogers et al., 2004a; Warrington, 1975).

The second group consists of patients who show multi-modal semantic impairments following left-hemisphere stroke affecting broad areas of inferior frontal and/or temporoparietal cortex. Jefferies and Lambon Ralph (2006), following Head (1926), have labeled this pattern *semantic aphasia* (SA). The central diagnostic criteria for SA include verbal and nonverbal comprehension impairments (as assessed, for example, by the word and picture versions of the Camel and Cactus Test) following left hemisphere stroke.

Several studies have now shown that patients with SA differ qualitatively from those with SD in several respects: (i) While non-verbal reasoning and executive functioning are preserved in SD, semantic impairments in SA correlate with the degree of executive dysfunction (Jefferies and Lambon Ralph, 2006). (ii) Itemwise consistency for the same concept across different tests is generally high in SD but much more variable in SA (Corbett et al., 2009a; Jefferies and Lambon Ralph, 2006). (iii) Across both verbal and nonverbal tasks, patients with SA benefit significantly from appropriate cueing and are disrupted by miscues (Corbett et al., 2011; Jefferies et al., 2008), whereas patients with SD are substantially less influenced by cueing. (iv) Whilst word frequency/concept familiarity strongly influences naming and comprehension in SD, patients with SA can show reduced or null effects of frequency/familiarity in naming (Hoffman et al., 2011; Jefferies and Lambon Ralph, 2006) and in some circumstances appear to show *better* comprehension of lower-frequency items (Almaghyuli et al., 2012). (v) While both groups produce semantic errors in naming, patients with SA produce many more associative errors (e.g., SQUIRREL→ “nuts”) and fewer “no response” errors compared to patients with SD (Jefferies and Lambon Ralph, 2006). (vi) Patients with SA are significantly more impaired when the task requires the participant to (a) match concepts that are only weakly related, (b) resolve conceptual ambiguity, or (c) correctly select a weak target from amongst several strongly competing distractors (Corbett et al., 2011; Noonan et al., 2010). Though these phenomena have been less well-studied in SD, Jefferies and Lambon Ralph (2006) found that task difficulty better predicted comprehension measures in SA than in SD.

To help understand the differences between patient groups, we here articulate a framework that builds on prior work both by our group (Jefferies, 2013; Jefferies and Lambon Ralph, 2006; Lambon Ralph, 2014; Patterson et al., 2007; Rogers et al., 2004a) and by others (Badre et al., 2005; Botvinick and Cohen, 2014; Duncan, 2010; Thompson-Schill et al., 1997; Wagner et al., 2001) that we will refer to as *controlled semantic cognition* (CSC). In common with many other theories, the CSC framework proposes that semantic knowledge involves the interactive activation of representations distributed throughout cortex that encode sensory, motor, linguistic and affective information (Martin and Chao, 2001; Meteyard et al., 2012; Pulvermüller, 2013). For instance, the concept “robin” draws on representations of this object's shape, color, texture, visual motion, sound and verbal associations. To this widespread general view the CSC framework adds two key components.

First, interactions amongst the various surface representations (“spokes”) are mediated by a domain-general cross-modal hub situated bilaterally in the anterior temporal cortex (Guo et al., 2013; Lambon Ralph, 2014; Mayberry et al., 2010; Patterson et al., 2007; Rogers et al., 2004a). While other pathways may also connect the various representations, the hub is critically important to semantic cognition because it allows the system to both learn and exploit conceptual similarity structure that is not directly captured by any single surface modality. Thus, for instance, we can discern that a stork and robin are similar kinds of things despite being quite different in shape, size, color, name and movement. This ability is fundamentally important for the generalization of acquired knowledge to novel items and situations, and the ATL supports such generalization by representing conceptually related items with similar patterns of neural activity (Rogers et al., 2004a).

Second, the CSC proposes that the “hub-and-spoke” network alone is insufficient to support successful semantic cognition in many situations. The network encodes a vast array of different features and associations, only a subset of which will be relevant in a given task context. To ensure that the “right” information comes to mind in a given situation, the flow of activation in the network is constrained by a representation of the current task context or goals (Jefferies and Lambon Ralph, 2006; Rogers and McClelland, 2004; Wagner et al., 2001). In this way the CSC framework draws upon the extensive literature on cognitive control, and in particular the “guided activation” approach to control (Botvinick and Cohen, 2014). In keeping with this literature, the CSC framework proposes that the task/goal representations are encoded within fronto-parietal networks, and that these representations help to generate task-appropriate responses by facilitating or “potentiating” interactions among sub-components of the hub-and-spokes network. Computational models consistent with this proposal have been described by Rogers and McClelland (2004), who showed how the central idea sheds light on a variety of phenomena in healthy cognition and cognitive development, and by Dilkina et al. (2008), who showed how variability across different semantic tasks in SD might arise within a system in which the flow of activation is constrained by representations of the current task.

### 1.2 Causes of semantic impairment in the CSC framework

In SD, semantic impairment is proposed to arise from neurodegeneration in the ATL hub, so that the pattern generated over the hub part of the semantic network in response to any given stimulus becomes increasingly distorted and the strength of interaction between the hub and “spoke” representations becomes increasingly muted (Guo et al., 2013; Lambon Ralph et al., 2007). In SA, the semantic impairment is held to arise from damage to the control system that shapes the flow of activation through the hub-and-spokes network, so that processing within the semantic network becomes pathologically noisy as the balance of activation and inhibition is thrown off-kilter—an effect that will be stronger for tasks or items that require a greater degree of control.

This proposal predicts certain similarities between the two syndromes. Simulations with neural network models of semantics have shown that a central proposed cause of dysfunction in each disorder—the distortion of hub representations in SD, and the pathologically noisy processing in SA—will produce similar effects: a loss of, or difficulty in activating, knowledge about properties that individuate semantic neighbors that is especially pronounced for lower-familiarity items (Rogers and McClelland, 2004; Rogers et al., 2004a, 2004b; Lambon Ralph et al., 2007). Individuating properties are especially vulnerable because, to retrieve them, the semantic representation at the hub must be specified very precisely. If the hub representation is distorted, either via direct hub damage or from noisy processing caused by degraded control, the item will be confused with its semantic neighbors and the ability to correctly activate its individuating properties will diminish. The effect is especially pronounced for lower-familiarity items, because their individuating properties are less robustly encoded and their representations are less well differentiated from semantic neighbors (see Rogers and McClelland (2004), for simulations). We refer to this cause of impairment, be it from noisy processing or damage to the semantic hub, as *representational distortion*.

Because the abnormality in SA is located in control systems, however, a second factor comes into play in this disorder: the magnitude of semantic impairment will vary with the control demands of the task and item. The factors governing recruitment of control have been well documented in studies of classic executive tasks, so it is possible to anticipate some of the conditions likely to impede semantic processing under this view. First, control is recruited when the participant must inhibit a prepotent response in favor of a less robust but context-appropriate response (e.g., the conflict condition of the Stroop task; Stroop, 1935). Second, more control is required when a target item or response must be discriminated or selected from among many other items, as is observed, for example, when the number of flankers increases in the inconsistent condition of the Eriksen flankers task (Eriksen, 1995). Third, more control is required when a target item must be discriminated from one or more very similar items, as when the flankers are spatially closer or are perceptually more similar to the target in the same task (Eriksen, 1995). In the context of semantic cognition, the CSC framework thus predicts that semantic deficits in SA will vary with the prepotency of the target response and with the number and similarity of competing items and responses in the task, in addition to effects of representational distortion due to noisy processing (see Corbett et al. (2009a, 2009b, 2011) and Noonan et al. (2010) for direct evidence of each of these factors in SA performance).

In what follows we compare and contrast the effects of familiarity, typicality, and specificity in the two groups across four common tasks. For each task, we describe the test design and take note of the factors that might govern impaired behavior across conditions according to the view we have just laid out. We then report the behavior of healthy controls and case series of patients with SD and SA on the tasks. Our aim is to assess the face validity of the CSC framework: does it offer a plausible account of the documented patterns? Following this report, we consider alternative accounts of the differences between SD and SA and how the current results bear on these. We conclude by considering the more general implications of the current results for theories about the neural bases of semantic knowledge.

## 2 Methods

### 2.1 Neuropsychological assessment and stimuli

Patients in both groups completed a typical selection of background neuropsychological assessments to confirm their diagnoses, as well as the Cambridge Semantic Battery (Bozeat et al., 2000) to establish the degree and multimodal nature of the semantic impairment for each patient (see Tables 1 and 2).

The principal novel contribution was the development and use of the *Levels of Familiarity, Typicality, and Specificity* (*LOFTS*) semantic battery. The central aim was to develop a set of items from a range of conceptual domains that would allow us to investigate independent effects of familiarity, typicality, and specificity in different semantic tasks. The items themselves and the details of the process by which they were selected are provided in Supplementary Materials.

The battery is comprised of two item subsets. The *Typicality* subset includes 16 triplets of items. Items within a triplet are matched for rated familiarity but vary in rated prototypicality, with each triplet containing one highly typical (rated 1–2 on a 7 point Likert scale), one moderately typical (rated 2–4.5) and one atypical item (rated above 4.5). These items thus allow us to assess effects of typicality unconfounded with familiarity. The *Specificity* subset contains 22 pairs of items, with each pair comprised of two recognizable subordinates of the same intermediate/basic-level category, one higher in rated familiarity and one lower. For instance, one pair includes two different varieties of cheese, one rated as highly familiar (cheddar) and one as less familiar (brie) (note that these assignments may be specific to the culture of the UK, where this research was conducted); another includes two varieties of large cats, one more familiar (lion) and one less (panther). All items were named by healthy controls with greater than 75% agreement. Thus this subset allows us to assess specific level naming and recognition and effects of familiarity on these abilities. Three pairs of items from the Specificity subset also appeared as items in the Typicality subset, so that the battery includes 86 items total. The items span a variety of categories, including animals, vehicles, tools, foods, and plants.

These LOFTS items were then used in semantic tasks that both do and do not require speech output: picture naming and category fluency for the former, word–picture matching and sorting for the latter. Section 3 is subdivided according to these tasks and so, in that section, we will describe each test before presenting the data.

### 2.2 Patient groups

The SD cohort consisted of 15 patients, though not all cases participated in every facet of the study: *N*'s per task varied from 10 to 14 and will be indicated in the corresponding section. Each case was initially seen by a senior neurologist/physician in UK hospital clinics in either Cambridge or Bath. Standard psychiatric rating scales were applied to exclude major psychiatric disorders such as depression and schizophrenia, and each patient also had structural brain imaging and the usual battery of screening blood tests to exclude treatable causes of dementia. All patients fulfilled the international consensus and local criteria for SD (Gorno-Tempini et al., 2011; Hodges et al., 1992; Neary et al., 1998), including impaired receptive and expressive content-word vocabulary and impoverished semantic knowledge with relative preservation of nonverbal reasoning, visuospatial abilities, phonology, syntax and day-to-day memory, and with MRI-confirmed focal atrophy in rostro-ventral regions of the temporal lobe. The majority of these cases have already been included in one or more previous publications on SD (for example, Patterson et al.,2006), so we have not provided detailed descriptions here. Demographic characteristics and some basic background neuropsychological data are given in Table 1.

The SA group consisted of 10 patients, recruited from stroke groups and speech and language therapy services in Manchester, UK. All of these patients have been described in previous papers (for example, Jefferies and Lambon Ralph, 2006; Noonan et al., 2010). Patients with chronic aphasia following a CVA at least a year previously were selected for inclusion in the study if they showed semantic impairment on both the picture and the word versions of a challenging 4AFC test of semantic association. These inclusion criteria were used by Jefferies and Lambon Ralph (2006) to assess whether patients who failed the same range of neuropsychological assessments despite differing aetiologies (CVA and SD) and distributions of brain damage would show qualitative differences in the pattern of semantic impairment. The SA patients were not specifically selected to show a deficit of semantic control, but nevertheless all of the cases showed clear effects of these manipulations in subsequent testing (e.g., Noonan et al., 2010).

The degree of semantic deficit ranged from moderately severe (e.g., cases LS and KA) to very mild (e.g., patient SC, who was within the normal range on the similar but easier 2AFC Pyramids and Palm Trees test; see data in Table 2). Five of the SA patients, who could be classified as cases of transcortical sensory aphasia, had relatively fluent speech and good repetition. The remaining cases had less fluent speech and/or poorer repetition (see Table 2 for details of aphasia classification from Boston Diagnostic Aphasia Examination). Table 2 also summarizes the lesion for each aphasic stroke patient. MRI was available for five cases (NY, SC, ME, KH, LS) and CT was available for a further two (BB, KA). It was not possible to obtain scans for three patients (PG, JM, MS) due to a lack of consent or contraindications for MRI, although written reports of previous CT scans were available for PG and JM. In line with the literature on semantic impairment in stroke aphasia, all of the patients had left temporoparietal- and/or frontal-lobe lesions.

### 2.3 Control participants

Performance on the tasks by the SA and SD patients was compared to a group of 12 neurologically-intact participants who were age- and education-matched to the patients.

## 3 Results

### 3.1 Task 1: picture naming

As noted earlier, both groups are anomic but with quite different profiles. In the first task we assessed picture naming with the LOFTS materials, with the aim of understanding how the patients' anomia is influenced by familiarity, typicality and specificity.

*Predicted effects of familiarity.* Prior work has distinguished SA from virtually all other forms of anomia, including SD, in the negligible impact of frequency/familiarity on SA naming accuracy. In the Cambridge 64-item naming task, for instance, the same SA cohort as in the current study showed about 33% correct naming of both higher- and lower-familiarity items, with no significant difference in accuracy. In the same study, patients with SD showed a highly reliable advantage in naming higher-familiarity items (Jefferies and Lambon Ralph, 2006). Hoffman et al. (2011) offered a potential explanation consistent with the CSC framework: more frequent words, because they occur in more diverse meaning contexts, become more polysemous and so place greater demands on control systems, which must help to resolve which meanings are relevant to a given situation. In the case of basic-level naming, for instance, an image of a highly familiar item may generate a pattern of activation in the hub which in turn begins to activate a representation of the corresponding basic-level name (“bird”). Because the word is high frequency, it is associated with a variety of meanings (for instance, “bird” as slang for “woman”). Thus re-entrant feedback from the word form to the hub may push the hub representation toward these other meanings, generating competition that must be resolved. Lower-frequency basic-level terms are associated with a narrower range of meanings, so the “echo” back from word-form to hub keeps the hub representation within the right neighborhood, generating less competition.

This explanation applies particularly to basic-level names which, as the default label, are applied across many different situations. More specific labels, in contrast, are applied within more restricted contexts, such as when the speaker wishes to refer to one among many exemplars of the same basic category (Wales et al., 1983). Thus specific labels should generally be less polysemous than basic-level items, and the control demands for specific naming of higher versus lower frequency items should be better matched—in which case, the same patients who show negligible familiarity effects when naming at the basic level should show significant effects for specific-level naming. Patients with SD should also show worse accuracy for specific naming of lower familiarity items, because these are less robustly encoded in the network, and because the corresponding representations are less well differentiated from their neighbors.

*Predicted effects of typicality.* In SD, loss of knowledge about properties that individuate semantic neighbors predicts a typicality advantage in naming, even when familiarity is controlled, since typical items share many properties with their neighbors and have few individuating properties (Rosch et al., 1976b). Accordingly, in a very large-*N* study of naming in SD, Woollams et al. (2008) have shown significantly better naming of more typical items even after partialling out the effects of frequency/familiarity.

What pattern is expected with degraded control? In SA, effects of representational distortion may be counteracted by control demands: by virtue of their similarity to other category members, typical items are likely to activate many related semantic representations, each with a different name. Atypical items will generate less competition because they are more distal to other category members (see Supplementary Materials). Naming may therefore require a greater degree of semantic control for more typical items, a factor that favors relative preservation of more atypical items in SA. The balance of representational distortion versus control demands is difficult to anticipate, but if anything the typicality advantage expected in SD, where control is not a major deficit, should be attenuated in SA.

*Predicted effects of specificity.* In both disorders, all relevant factors predict worse naming at the specific level than in the standard basic-level naming task: specific-level names are generally less frequent, require individuation of closely related items, and demand greater control since they are not the “automatic” names produced across many contexts.

### 3.1.1 Participants

Ten of the 15 patients with SD completed the naming task. Assessment of the more seriously impaired patients was abandoned after one such patient was unable to complete the task. Likewise, naming was not attempted in the three SA cases with severe expressive aphasia.

### 3.1.2 Procedure

Participants named colored photographs of the Typicality and Specificity subsets in two different sessions. Participants who produced correct responses more general than the target (e.g., “bird” instead of “robin”) were prompted for a more specific name by asking “Do you know what kind it is?” Responses were scored as correct if the same name had been produced by at least two controls in the original item-screening study. Incorrect responses were classified as one of the following error types: level error, reflecting an overly-general response (e.g., DALMATIAN→ “dog” or “animal”; DOG→ “animal”), semantic error, circumlocution, phonological error or no-response. Where the respondent self-corrected, the recorded response was the final item generated. Any other response, such as the production of a name with no clear relationship to the target, a nonsense utterance, or an associative error, was classified as “other”.

### 3.1.3 Results

The SD cohort was divided into two groups based on a median split. The milder group was closely matched to the SA group in naming accuracy on the standard Cambridge battery.

**Fig. 1:**
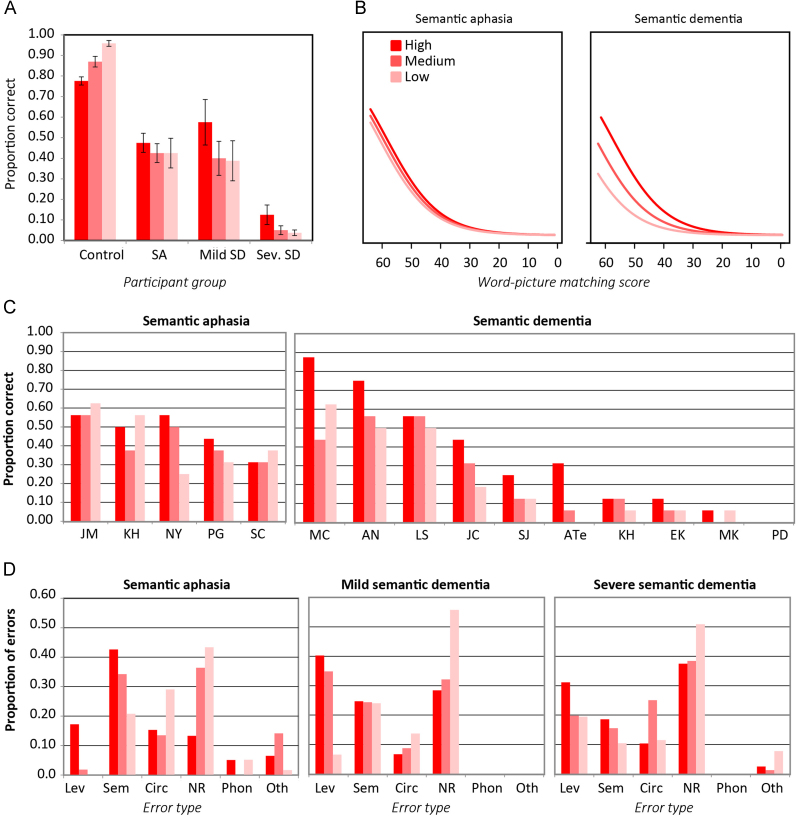
Naming performance for the Typicality subset. (A) Mean and standard errors of accuracy (proportion correct) for controls and patient cohorts at different levels of typicality. (B) Predicted accuracy from a logistic mixed effects model. (C) Individual patient accuracy for each level of typicality. (D) Error distributions by typicality for each patient group. Incorrect responses are categorized as level errors (LEV), reflecting an overly-general response (e.g., DALMATION→ “dog”), semantic errors (SEM), circumlocutions (CIRC), no-response (NR), responses that are phonologically related to the target (PHON) and other errors.

Fig. 1A shows the mean and standard error of the accuracy for each group in naming the Typicality subset. Controls performed better for more atypical items, whereas patients with SD showed the opposite effect. The SA cohort, in contrast to both controls and SD, was unaffected by typicality. Accordingly, a paired-samples *t*-test comparing accuracy on the highest and lowest typicality items showed reliably different accuracy for the mild SD cohort (*t*=4.74, df=4, *p*<0.01) but not the SA cohort (*t*=0.67, df=4, *p*=n.s.).

Effects of typicality across the two groups were compared with a logistic mixed effects model (see Supplementary Materials). Fig. 1B shows expected accuracies generated from the fitted model, while Fig. 1C shows the data for individual participants. In addition to the expected effect of semantic severity on naming accuracy, the analysis revealed no difference between groups in the overall degree of anomia and no main effect of typicality, but a marginally reliable interaction between patient group and typicality (*B*=−0.51, *Z*=−1.95, *p*<0.052), with typicality exerting a larger effect on accuracy for the SD than the SA group.

Finally, Fig. 1D shows the distribution of error types as a proportion of all errors, calculated separately for high-typical, medium-typical, and low-typical items, across each patient group. To assess whether the distribution of error types differed across patients with a comparably severe anomia, we computed a *χ*^2^ statistic comparing the error frequency across types in the SA and mild SD cohorts. The two groups showed reliably different patterns of errors (*χ*^2^=39, df=4, *p*<0.001), and inspection of Fig. 1D illustrates why. Consistent with prior work, patients with milder SD made frequent no-response errors, especially on the lowest-typicality subset, and fairly frequent level errors, especially on high- and mid-typicality targets. They produced a moderate number of semantic and circumlocution errors, and no phonological or other errors. The SA cohort showed the reverse pattern: relatively more semantic and circumlocution errors, relatively fewer level and omission errors, and a small but non-zero number of phonological and other errors. In more advanced SD cases, the pattern is fairly similar to the milder sub-group, though a couple of “other” errors have crept in.

**Fig. 2:**
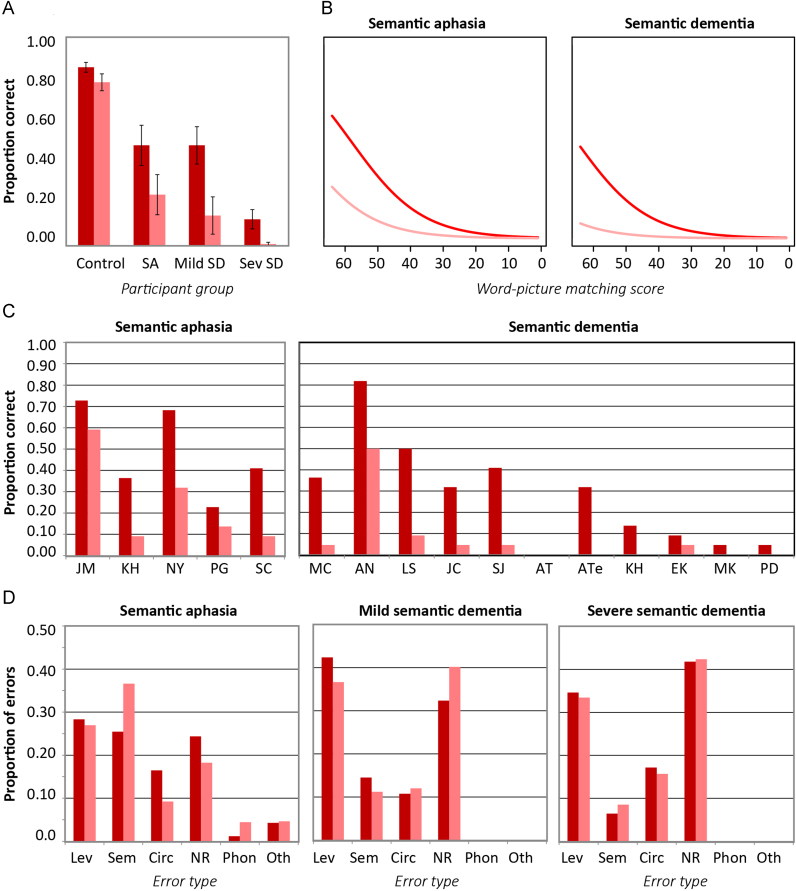
Naming performance for the Specific subset. (A) Mean and standard errors of accuracy (proportion correct) for controls and the patient cohorts for lower and higher familiarity items. (B) Predicted accuracy on the Specific subset from a logistic mixed effects model. (C) Individual patient accuracy for lower and higher familiarity items. (D) Error distributions by familiarity for each patient group. Incorrect responses are categorized as level errors (LEV), reflecting an overly-general response (e.g., DALMATIAN→ “dog”), semantic errors (SEM), circumlocutions (CIRC), no-response (NR), responses that are phonologically related to the target (PHON) and other errors.

Fig. 2A shows the mean and standard error of naming accuracy for the Specific subset. Controls were marginally less likely to produce a correct response for lower-frequency than higher-frequency items (paired-samples *t*=1.93, df=11, *p*<0.08). This effect was greatly amplified in all three patient cohorts. Notably, whereas prior work has shown little or no frequency effect in basic-level naming in SA (Jefferies and Lambon Ralph, 2006), a reliable frequency effect was observed in this task (paired-samples *t*=4.49, df=4, *p*<0.02). As expected, a highly significant frequency effect was observed in the mild SD cohort (paired-samples *t*=14.41, df=4, *p*<0.001).

Effects in the two groups were again compared with a logistic mixed effects model (see Supplementary Materials). Expected behavior from the model is shown in Fig. 2B, while the data for individual cases is shown in Fig. 2C. In addition to the expected effect of semantic impairment severity, the model revealed a reliable effect of item frequency with worse performance for low-frequency items (*B*=−1.56, *Z*=−2.72, *p*<0.007), but no effect of group and no interaction between group and frequency. Thus in contrast to prior results from basic-level naming, frequency appears to have a similar impact on specific-level naming in the two patient groups.

Finally, we again compared the distribution of error types across the two patient groups using a *χ*^2^ test on the count of errors across types, summed across all participants in each patient group. The probability of each error type, computed separately for high- and low-frequency items, is shown in Fig. 2D. The distribution again differed significantly from chance (*χ*^2^=73, *p*<0.0001), with a similar profile to that observed on the Typicality subset: relative to SA, patients with SD made more level and no-response errors, fewer semantic and circumlocution errors, and no phonological or other errors.

### 3.1.4 Summary

The results are generally consistent with the CSC framework. Patients with SD showed a typicality advantage in naming that was especially striking in contrast to controls, who showed the reverse effect. No typicality effect was observed in patients with SA, consistent with the view that the typicality advantage arising from noisy processing is attenuated by the greater competition elicited by typical items. With regard to familiarity, patients with SA showed frequency effects for specific-level naming equal in magnitude to those observed in SD, in contrast to prior results of basic-level naming. This might be anticipated by the CSC framework since specific labels are less polysemous than basic-level items, and thus competition is minimized. We also note that specific-level naming was substantially worse than standard basic-level naming for all patients, though this is not surprising given that all factors mediate toward such a result. Finally, consistent with prior work, the two groups showed quite different distributions of naming errors.

### 3.2 Task 2: picture sorting

In the second task participants were asked to sort pictures of the LOFTS items into three either very general semantic categories (animal, plant, manmade object) or three more specific categories (e.g. for animals: land, air, water creature), across two different sessions. The same items were used in the specific and general sorts, allowing us to estimate effects of specificity unconfounded with familiarity. Relative to naming, uncertainty about the size or nature of the response set was eliminated because this set (i.e., the three sorting categories) is explicitly specified. Moreover, the task requires no verbal response, so, in contrast to naming, patterns of impairment cannot be directly attributed speech production deficits. Finally, though the task manipulates the specificity of the sorting categories, even the more specific condition involves discerning more general distinctions than those required by naming. What then are the relevant factors for understanding familiarity, typicality, and specificity in this task?

*Predicted effects of familiarity.* The CSC framework predicts that, in contrast to naming, familiarity effects should be reduced or eliminated in this task in both disorders. Such effects arise in naming because highly familiar items are better differentiated from their immediate semantic neighbors—for instance, representations of dogs will be better-differentiated from those of wolves, foxes, and coyotes. Thus lower-frequency items are more likely comingle with representational distortion. Regardless of their individual frequency, however, all such four-legged land animals will be equally well differentiated from more distal concepts, such as various birds, fish, vehicles, and so on. Thus frequency/familiarity should have a strong effect when the task requires individuation of an item from its close neighbors, as in naming, but not when the items are to be differentiated from more distal concepts, as in sorting.

*Predicted effects of typicality.* As already noted, highly typical items share many properties with their semantic neighbors and possess few individuating properties—thus such items are proximal to their neighbors and distal to items from different categories. Atypical items are more distal to members of the same category, but more similar to members of other categories—for instance, jellyfish, because they lack eyes, fins, tails, and so on, are less similar to other marine animals and more similar to plants (see Supplementary Materials). Thus atypical items are more likely than typical items to be confused with members of a neighboring category when representations are distorted. Of course, such effects will be greatly reduced when the sorting categories are very semantically distinct. Thus representational distortion predicts a typicality advantage, especially for the more specific sorting task. Control demands likewise favor typical items in this task: though such items activate many similar representations, all such items will belong to the same sorting class and so will bring about a similar response, producing little competition (similar to the consistent conditions of classic control tasks). Atypical items likely activate fewer semantic representations overall, but are more likely to activate items from different response categories, thus generating more competition, especially for more specific sorting. Thus both representational distortion and control demands predict worse performance for atypical items that is most pronounced for specific-level sorting.

*Predicted effects of specificity.* Representational distortion will produce worse performance in the more specific condition, since the different sorting categories are semantically more proximal in this condition. With regard to control demands, some relevant factors are matched across conditions: the number of response options is held constant, and the set of semantic representations activated by a given item support the same sorting response and so generate little competition as already noted. The main factor likely to influence behavior again arises from semantic proximity of the responses: options are more similar in the Specific condition, so if anything control demands should be higher (and performance worse) in this condition. Thus disordered control also favors greater impairment of more specific sorting.

In summary, the two groups are expected to look qualitatively similar, showing a null or attenuated effect of familiarity, a typicality advantage especially for specific sorting, and an advantage for general sorting.

### 3.2.1 Participants

The task was completed by the same age-matched controls as previously, by 9 patients in the SA cohort and all but one of the patients in the SD cohort.

### 3.2.2 Stimuli

All items from the two subsets were administered except for a small number of food items that did not fit the more specific sorting categories; an additional set of filler items was added to the set to balance the number of items appearing in each category. Performance on these items was not considered when computing accuracy scores.

### 3.2.3 Procedure

The task was administered over two testing sessions on different days. In the first session, participants were asked to sort the pictures into three categories corresponding to animals, plants or manmade objects. Printed category labels were placed on the table facing the participants and were read aloud on each trial. In the second session, participants were asked to sort the animals into land, water, or air creatures; the plants into trees, flowers, or fruits/vegetables; and finally the manmade objects into vehicles, clothing, or tools.

### 3.2.4 Results

**Fig. 3:**
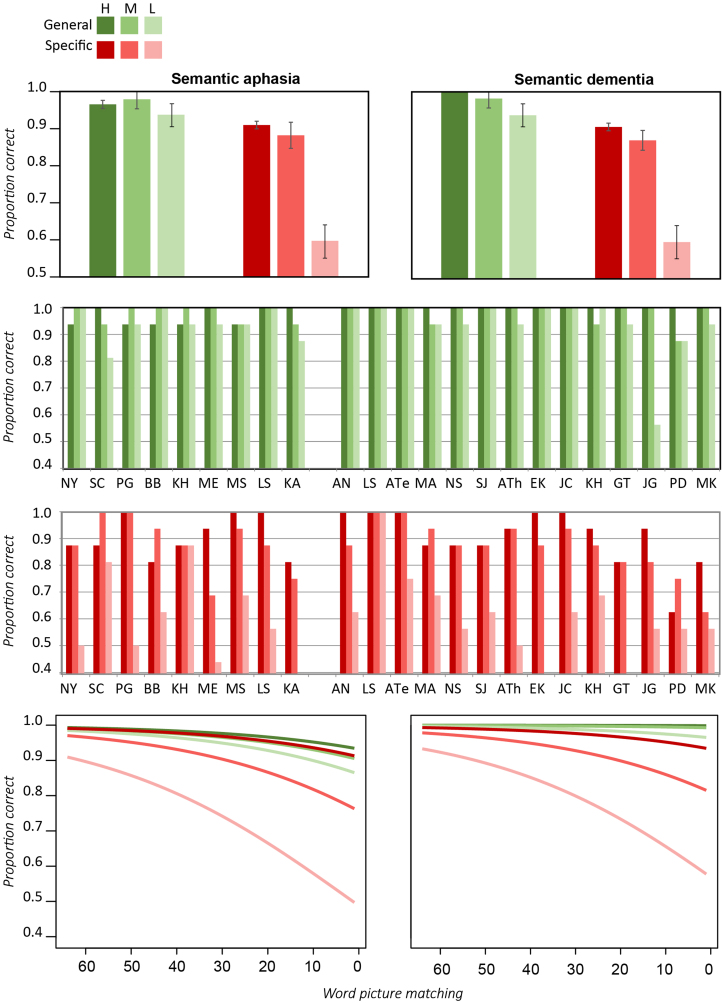
Picture sorting at general (green) and specific (red) levels for the Typicality subset; more typical items are depicted with more saturated colors. Top: mean and standard errors of accuracy for patient cohorts at each level of typicality. Middle: individual accuracy at each level of specificity for general and specific levels. Bottom: predicted accuracy from a logistic mixed effects model. (For interpretation of the references to color in this figure legend, the reader is referred to the web version of this article.)

Control performance was near ceiling and was not investigated further. Fig. 3 shows sorting accuracy as a function of level (general or specific) in each patient cohort for the Typicality subset. In both cases, performance was better for the general than the specific sort, and in both cases accuracy was better for more typical items, especially at the more specific level. These observations from group means were also mirrored in the individual patient data.

Logistic mixed effects modeling was again used to compare effects across groups (see Supplementary Materials). As Fig. 3 makes clear, a qualitatively similar pattern was observed across groups, with both groups showing a typicality advantage that is much more pronounced for more specific sorting. The mixed effects model revealed, however, that the magnitude of the effects differed reliably between groups. For the general sorting condition, overall performance was better for the SD than the SA group (*B*=3.84, *Z*=2.62, *p*<0.009), and across groups a reliable effect of typicality was observed for specific but not general sorting (*B*=−0.78, *Z*=−2.27, *p*<0.03). The effects of level and typicality, and the interaction between these, all themselves interacted with patient group. In the general sort, the effect of typicality was larger in the SD than in the SA group (*B*=−1.19, *Z*=−2.177, *p*<0.03), a result that can also be observed in the individual patient plots and means. The difference in accuracy between general and specific levels was also significantly larger in the SD than the SA group (*B*=−3.54, *Z*=−2.27, *p*<0.03). Finally, patients with SD showed a larger effect of typicality than those with SA at the general level (*B*=−1.19, *Z*=−2.177, *p*<0.03), but a smaller effect at the specific level (*B*=1.2, *Z*=2.03, *p*<0.05). In summary, while typicality exerts a larger influence on specific than on general sorting in both groups, these influences are more similar across levels in SD, while the effect of specificity is more pronounced in SD than in SA.

We also considered whether the poor performance for low-typicality items in both groups arose because the correct answer for some items might be ambiguous when sorting at the specific-level. For instance, penguins might be considered either land or water animals, leading to uncertainty that might be exacerbated in patient participants. From the control data, we identified six items where controls showed less than 80% agreement as to the specific-level category (frog, crocodile, penguin, swan, duck and bat). Removing these items left the pattern of results unchanged: both patient groups showed equivalently good performance on high and medium typicality items (91% correct at both levels in both groups) but very significant impairments for low-typicality items (69% correct for SA, 64% for SD, as compared to 98% correct for controls). Thus poor sorting of atypical items occurs even for items whose specific-category membership is unambiguous to controls.

**Fig. 4:**
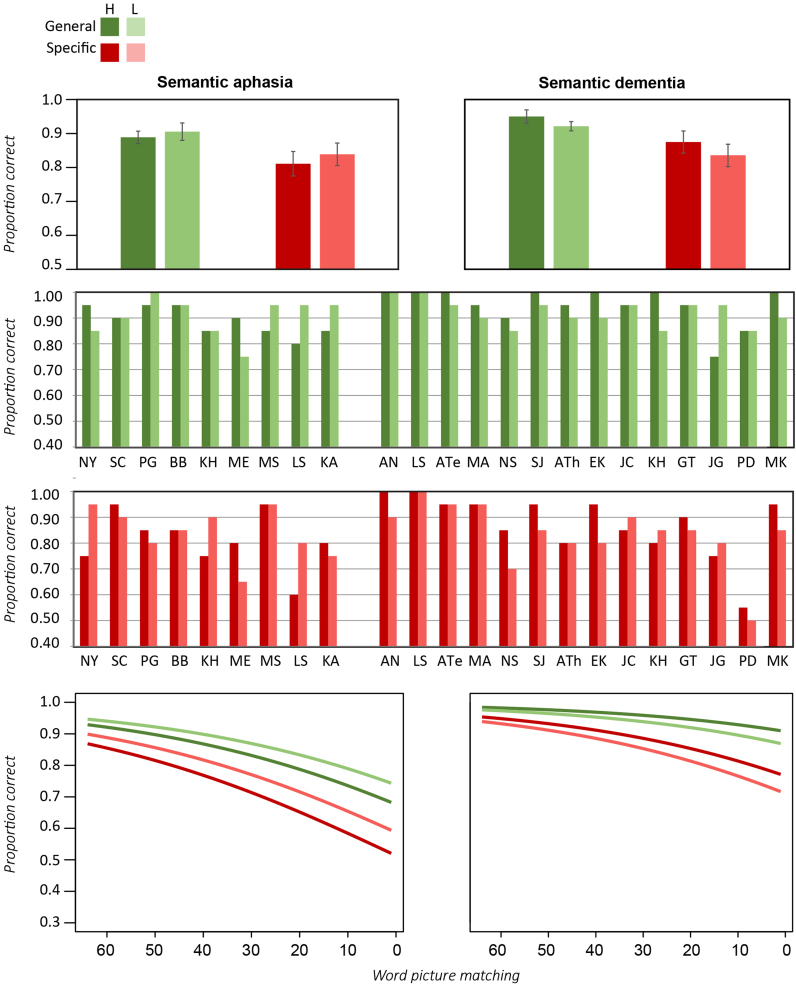
Picture sorting at general (green) and specific (red) levels for the Specific subset; higher-familiarity items are depicted with more saturated colors. Top: mean and standard errors of accuracy for patient cohorts at each level of familiarity. Middle: individual accuracy at each level of familiarity for general and specific levels. Bottom: predicted accuracy from a logistic mixed effects model. (For interpretation of the references to color in this figure legend, the reader is referred to the web version of this article.)

Fig. 4 shows sorting accuracy for the Specificity subset as a function of level, familiarity and patient group. In both groups, means show little difference between higher and lower familiarity items but worse performance for the more specific condition. Expected performance from a logistic mixed effects model (see Supplementary Materials) is shown in the bottom row of Fig. 4. The SD cohort performed better than the SA patients at the more general sort (*B*=1.54, *Z*=3.34, *p*<0.001) and in both patient groups performance was reliably worse for the more specific sorting task (*B*=−0.68, *Z*=−2.20, *p*<0.03). No effect of item familiarity was observed nor was the interaction of any model variable with patient group significant.

### 3.2.5 Summary

The results are again consistent with the CSC view. Whereas naming showed a significant familiarity advantage in both groups, no familiarity effect was observed at either level for sorting. Whereas naming showed different sensitivity to typicality across groups, both groups showed the same pattern in sorting: a typicality advantage for more specific sorting that was attenuated at more general sorting. Finally, both groups showed worse performance for the more specific sort, especially for less typical items. The patterns were qualitatively similar in the two groups, though statistical analysisshowed that the magnitude of the effects differed reliably , with specificity exerting a stronger effect in SD, and typicality exerting a more similar effect across levels in SD.

### 3.3 Task 3: word–picture matching varying the semantic distance of foils

In the third task, we used word–picture matching to assess comprehension of the LOFTS items while varying the precision with which the concept must be understood. Across conditions, the same probe image appears but the distractor pictures vary in their semantic distance from the target. For instance, the probe word “cheetah” is paired with the same target picture, once with other cat pictures as distractors, once with non-feline mammals, once with non-mammal animals, and once with non-animals. The probe word is always a specific or basic level name, so the task always requires the participant to resolve the word and image meanings sufficiently well to discriminate these from semantic neighbors. This contrasts with picture sorting, where even the more specific sort could be accomplished through relatively coarse semantic distinctions. Thus the view we have developed predicts somewhat different effects of familiarity, typicality, and specificity in this task.

*Predicted effects of familiarity.* More familiar items are better differentiated from their neighbors, leading to greater preservation especially of more specific names and attributes when representations are distorted. Control demands likewise ought to favor more familiar items: first, more familiar names are likely to be more prepotent, and second, specific names are less likely to be highly polysemous, as argued above for naming. Thus in contrast to sorting, both groups should show a familiarity advantage.

*Predicted effects of typicality.* Relative to typical items, atypical items are both more distal to members of their own categories and more similar to members of other categories. Thus when representations are distorted, atypical items are less likely than typical items to be confused with closer distractors, but more likely to be confused with more distal distractors. Control demands will be similarly affected: typical items will activate representations of many semantic neighbors, each possessing a different name, and so will generate competition that is especially fierce when distractors are close. Atypical items are more likely to compete with items from neighboring categories and so will elicit greater control demands than typical items when distractors are more distal. Thus both representational distortion and control demands predict an interaction between typicality and distractor proximity, with a typicality advantage for more distal but not more proximal distractors.

*Predicted effects of specificity.* Representational distortion produces confusion of semantically related items, an effect that favors better performance when distractors are semantically more distal. Although the number of distractors in each array is held constant across levels, control demands are nevertheless likely to be higher for more proximal distractors since these are more similar to the target and hence generate a greater degree of competition. Thus representational distortion and disordered control both predict worse performance for more proximal distractors.

In summary, the CSC framework again predicts qualitatively similar effects in the two groups: better performance for more familiar items and for more distal distractors, and an interaction between typicality and distractor proximity, with typicality favouring performance when distractors are more distal but not when they are more proximal.

### 3.3.1 Participants

Participants were the same as in the sorting task.

### 3.3.2 Stimuli

Stimuli were arrays of color photographs, each with a target item and six distractors. The arrays were organized into four subsets varying in the semantic distance between the target and distractor items, which could be very close (same basic category), close (same intermediate category), distal (same superordinate domain) or unrelated (different superordinate domain). For items in the Specific subset, each photograph appeared once as a target at each level of specificity. For items in the Typicality subset, each photograph appeared once with close distractors and once with distal distractors. Target and distractor images appeared in approximately 5 arrays on average so that overall familiarity would not serve as a cue to the correct target. Further details are provided in Supplementary Materials.

### 3.3.3 Procedure

The test was administered in four blocks, each carried out on a different day. For each trial, the tester showed the array of images to the participant, read the word printed at the top of the page, and asked the participant to point to the item that matched the word. Patient participants completed all four testing blocks. Healthy controls completed a single block of testing, using only the most proximal distractors for each item, as it was anticipated that performance would be at ceiling for other proximity levels.

### 3.3.4 Results

**Fig. 5:**
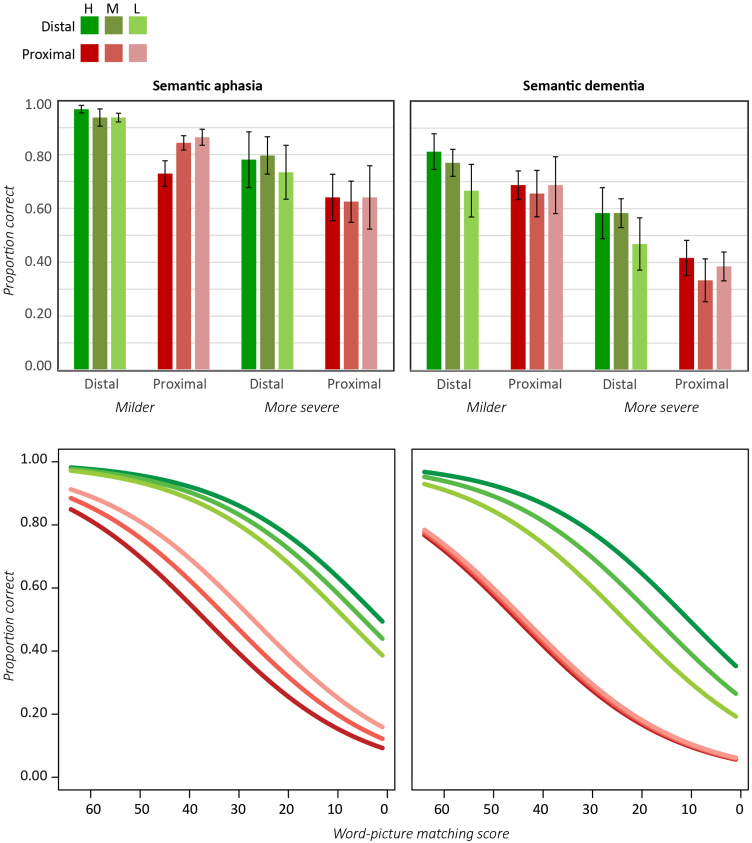
Word–picture matching performance with distal (green) and proximal (red) distractors for the Typicality subset; more typical items are shown with more saturated colors. Top: Mean and standard error of accuracy (proportion correct) for each level of typicality and distractor distance, for the semantic aphasia (left) and semantic dementia (right) cohorts. Bottom: predicted accuracy from a logistic mixed effects model. (For interpretation of the references to color in this figure legend, the reader is referred to the web version of this article.)

Fig. 5 shows performance of each patient group for the Typicality subset, as a function of semantic severity, the semantic distance of the distractors and item typicality. Individual patient data, though not shown, align well with the aggregate data. In both groups accuracy was worse for more proximal distractors, but the effect appeared to interact with typicality in both groups: for proximal distractors, increased typicality either reduced performance (milder SA) or at least failed to help it (SD and more severe SA), but distal distractors showed a typicality advantage.

Expected performance from a logistic mixed effects model (see Supplementary Materials) is shown in the bottom row of Fig. 5. Consistent with the preceding observations, the analysis revealed a reliable effect of impairment severity (*B*=0.06, *Z*=5.35, *p*<0.001) and of semantic distance, with worse performance for closer distractors (*B*=2.25, *Z*=4.22, *p*<0.001) and no difference between groups. No main effect of typicality was observed, but typicality interacted significantly with semantic distance, in the same way across patient groups (*B*=−.53, Z=−2.17, *p*<0.04). Fig. 5 shows why: more typical items elicited lower or equivalent accuracy when distractors were close neighbors, but higher accuracy when distractors were more distal. Overall performance did not differ between patient groups, nor did group interact reliably with any other variable.

**Fig. 6:**
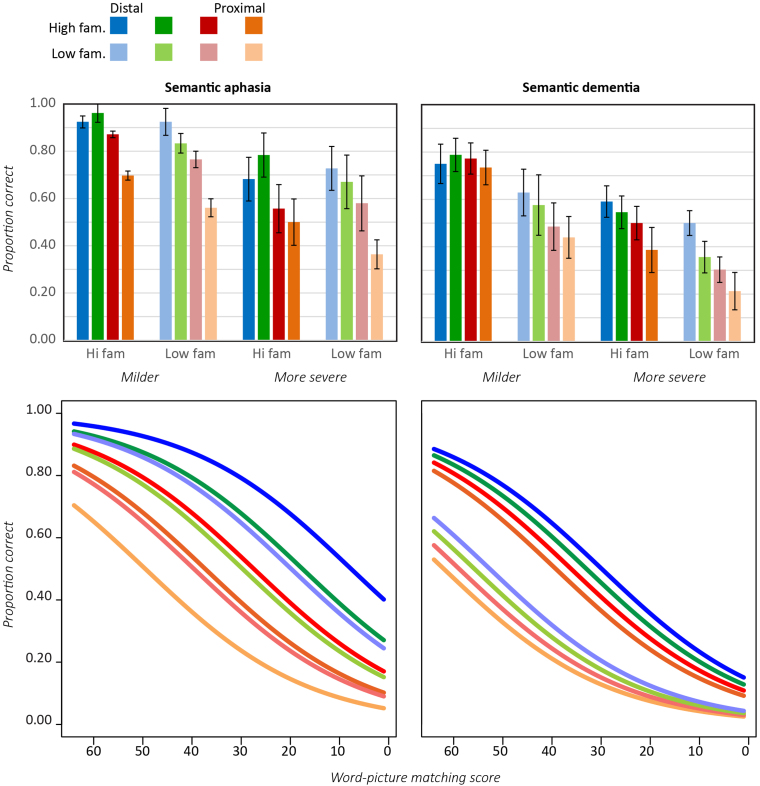
Word–picture matching performance for the Specific subset, showing accuracy as a function of distractor distance (colder colors=more distal distractors, warmer colors=proximal distractors) and item familiarity (more saturated=higher familiarity). Top: mean and standard errors of accuracy (proportion correct) by distance, familiarity, and patient group. Bottom: predicted accuracy from a logistic mixed effects model. (For interpretation of the references to color in this figure legend, the reader is referred to the web version of this article.)

Fig. 6A shows accuracy for the Specific subset, which was probed across four levels of distractor, from very close (same basic level category) to very distal (different conceptual domain). In both groups accuracy grew with the distance of the distractors. The effect appeared more pronounced in the SA than the SD cohorts, however. Both groups performed worse for less familiar items, but in this case the effect appeared more pronounced in the SD group.

Fig. 6B shows expected performance from a logistic mixed effects model (Supplementary Materials). Accuracy was reliably influenced by the severity of the impairment (*B*=0.06, *Z*=4.81, *p*<0.001), the distance of the distractors (*B*=0.59, *Z*=6.80, *p*<0.001), and the concept familiarity (*B*=−0.73, *Z*=−2.17, *p*<0.03) across both groups, with the SA cohort showing a large effect of distance and a modest effect of familiarity while the SD cohort showed a smaller effect of distance (*B*=−.41, *Z*=−3.71, *p*<0.001) and a larger effect of familiarity (*B*=−.63, *Z*=−2.44, *p*<0.02).

### 3.3.5 Summary

The results are qualitatively consistent with the CSC framework: both groups showed a reliable advantage for more familiar items (in contrast to sorting) and for arrays with more distal distractors, as well as the expected interaction between typicality and semantic proximity. As in sorting, the analysis also revealed reliable differences in the magnitude of these effects across groups, with the SD cohort showing a larger effect of familiarity and a smaller effect of proximity relative to the SA cohort.

### 3.4 Task 4: category versus letter fluency

Tasks 2 and 3 can be viewed as minimizing or holding constant some of the factors that influence demand for cognitive control. In the final study we considered data from verbal fluency, a task that draws on several cognitive abilities, including semantic and phonological knowledge, and is also known to strongly tax executive systems. Like naming, the participant must decide which of many possible competing responses to produce and must generate the correct phonological form of each item, but further demands on control also arise: the participant must maintain an effective mental search for possible responses, compare candidate responses to the fluency criterion and inhibit inappropriate items, monitor performance, remember prior responses and use this memory to avoid repetition.

Though verbal fluency draws simultaneously on many cognitive abilities, prior work has shown that different neuropsychological impairments can produce contrastive patterns of dysfunction across different variants of the task. For instance, patients with dysexecutive symptoms and generally fluent speech exhibit considerable and equivalent degrees of impairment in letter-fluency and category-fluency, whereas patients with semantic dementia generally perform worse at the latter than the former (Hodges et al., 1992). Thus, comparing patterns of deficits across different fluency tasks can shed light on the underlying causes of impairment. Specifically, fluency for semantic categories (e.g., animals, vehicles) requires the participant to generate a set of semantically related words, whereas fluency for phonological/orthographic categories (e.g., “words beginning with F”) does not draw significantly upon knowledge of semantic structure, and in fact may require suppression of semantic associates that spring to mind. Both tasks share the executive components noted above, but category fluency additionally requires knowledge of semantic structure and letter fluency has additional control demands. What then should fluency impairments look like in the two disorders?

*Typicality and familiarity.* Because the fluency task is open-ended, we could not experimentally vary the typicality or familiarity of the items produced.

*Predicted effects of specificity.* The effect of specificity on verbal fluency was investigated by comparing performance on two more general categories (animals, vehicles) versus two subordinates of these (dogs, boats). General categories permit production of items that are more semantically distal, whereas more specific categories require production of items that are all semantically related. Thus representational distortion should produce greater impairment for more specific categories in SD. Both general and specific fluency place considerable demands on control processes, however, so disordered control should lead to very poor performance in both conditions, potentially attenuating any effect of specificity in SA.

*Assessing the role of cognitive control in SA and SD.* Finally, the contrast of semantic and letter fluency may shed light on the proposal that semantic dysfunction in SA arises from disordered control. If fluency impairments are mainly caused by disordered control in SA, the impairments should be equally severe regardless of the particular fluency task, since the tasks share many of the same control demands. In contrast, if the impairments in SD are caused by distortion of semantic representations, these should be more pronounced in category than letter fluency, since the former explicitly requires knowledge of semantic structure. This view thus suggests that (a) category and letter fluency should be equally impaired in SA, (b) letter fluency should be less impaired than category fluency in SD (as has been often observed, e.g. Hodges et al., 1992) and (c) patients with SA should be more impaired than patients with SD on letter fluency, but not category fluency. Finally, the nature of the errors should differ between groups. Specifically, patients with SA should produce more incorrect responses in all tasks, as they have difficulty inhibiting either inappropriate associates or prior responses.

### 3.4.1 Participants

In the SD cohort, all participants completed the category-fluency task and all but two (KH and JG) completed the letter fluency task. In the SA cohort, all bar the most severely impaired participant (KA) completed both tasks. Sixteen healthy controls age-matched to the SD cohort completed the two tasks.

### 3.4.2 Procedure

For each fluency task, participants were given one minute to list as many items as they could think. Fluency data were collected for two general semantic categories (animals, vehicles), for two specific categories that are subordinate to the same general categories (birds, boats), and for the letters F, A, and S.

### 3.4.3 Results

**Fig. 7:**
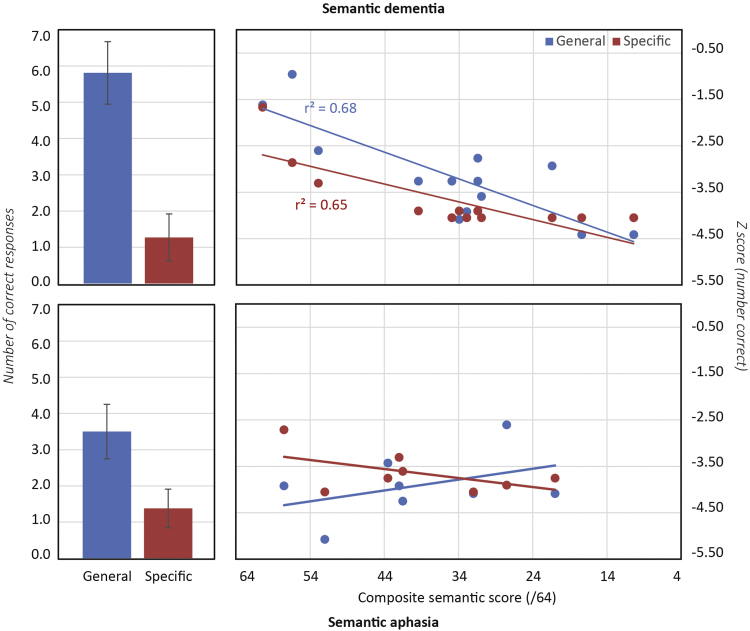
Performance on the category fluency task for more general and more specific categories. Left panels show mean and standard errors of the number of correct responses generated in each condition by patient group. Right panels show the relationship between the composite measure of semantic impairment and the number of correct responses generated in each condition, normalized by the mean and standard deviation of the control data. Linear fits in each condition are shown; where the simple correlation was statistically significant, *r*^2^ values are shown.

Fig. 7 (left) shows the mean number of correct responses generated for general and specific semantic categories, collapsed across all patients in each group. Both groups produced fewer correct items for specific relative to general categories, though the relative magnitudes of the impairments are hard to assess because controls also showed this pattern [mean (standard deviation)−general categories=15.4 (3.0); specific categories=13.6 (3.4): *p*<0.03 paired-samples *t*-test]. The right panels thus show *Z*-scored performance plotted against the magnitude of semantic impairment estimated from standard tasks. In SD performance is worse for specific than for general categories, and declines with the severity of the semantic impairment. The pattern in SA is different: performance does not reliably decline with increasing semantic impairment but is poor across the board, and is equally poor for general and specific level categories.

These observations were tested with a linear mixed effects model (see Supplementary Materials). Overall severity of the semantic impairment was found to improve model fit for SD but not the SA group (*χ*^2^=9.4, *p*<0.003). Model fit did not reliably improve when level of specificity was added to this model as a simple effect, but addition of a term for the interaction of specificity with patient group did reliably improve model fit (*χ*^2^=5.5, *p*<0.02), indicating different effects of specificity in the two groups. Together the analysis suggests that (a) degree of impairment in the task correlates with degree of semantic impairment in the SD group only, and (b) performance is reliably worse for more specific categories in the SD but not the SA group.

**Fig. 8:**
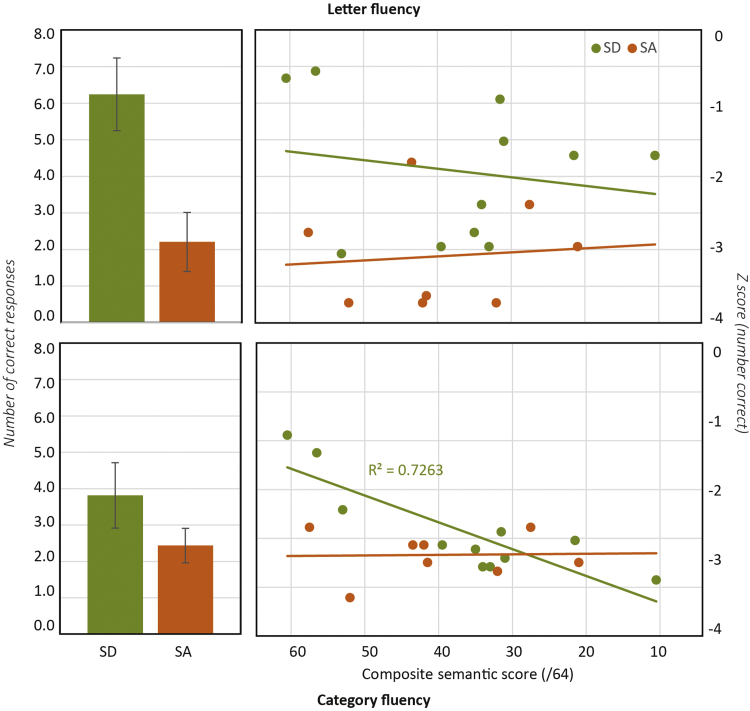
Comparison of performance on the category fluency task (across all categories) and the letter fluency task (across the letters F, A and S). Left panels show means and standard errors of the number of correct responses generated for each patient group on each task. Right panels show the relationship between the composite measure of semantic impairment and the number of correct responses generated, normalized by the control mean and standard deviation in each condition. Linear fits between these variables are shown; where the simple correlations are stronger than expected by chance, *r*^2^ values are also shown.

Fig. 8 (left) shows the mean number of correct responses generated per category across the letter (top) and semantic fluency (bottom) tasks, collapsed across all patients in each group. The effects of patient group, task type, and severity of the semantic impairment were again assessed with a mixed linear model predicting standardized performance (*Z*-scores of number of correct items generated). We found no effect of the composite semantic score on its own, but a reliable interaction between this score and task type (*χ*^2^=7.2, *p*<0.01), indicating that semantic severity exerted different effects on the two fluency tasks. Similarly, patient group did not improve model fit on its own but interacted reliably with task type (*χ*^2^=3.7, *p*<0.05), with a larger discrepancy between tasks for the SD than the SA group. The interaction between the composite semantic score and patient group was not reliable but the three-way interaction amongst this score, patient group and task type marginally improved model fit (*χ*^2^=3.32, *p*<0.07). Thus the mixed model analyses support the impression from Fig. 8: the difference between letter and category fluency is larger in SD than SA, and the magnitude of the semantic impairment predicts performance in the category but not the letter fluency task, more so in the SD cohort.

We next considered the error rates across the two groups, measured as the proportion of responses that were incorrect either by virtue of being repetitions of previous correct responses, or not meeting the fluency criteria. In category fluency, patients with SD produced errors at a rate comparable to controls (5% errors for SD in both general and specific cases, 3% for controls in both cases). Patients with SA, in contrast, were very much more likely to produce incorrect responses (44% for specific, 35% for general, a specificity-difference that is not reliable by a 2-tailed paired-samples *t*-test). Collapsing across general and specific levels, the difference between patient groups was highly significant (*p*<0.0001, 2-tailed between-samples *t*-test).With regards to letter fluency, patients with SA were again significantly more likely to produce incorrect responses (34% errors) than were patients with SD (8% errors; *p*<0.0001 two-tailed between-samples *t*-test).

Finally, we considered the nature of the errors produced in SA across category and letter fluency tasks. The great majority were of three kinds: (1) repetitions, in which a correct but previously-generated item was produced, (2) prior-task perseverations, in which an item from a previously-administered fluency task was produced and (3) “semantic drift” errors in which the participant began with an appropriate item but followed it with a chain of semantic or thematic associates. For instance, for the category “birds”, one participant generated the sequence “parrot, monkey, koala, cat, dog”, while for the letter F, another participant generated the responses “Fred, Phyllis, Marion, Amanda, Bob, Fred”.

### 3.4.4 Summary

The observations from verbal fluency are again consistent with the CSC framework: Specificity influenced performance in SD but not SA, and letter fluency was more impaired in SA than SD even though the two groups showed equivalent impairment for category fluency. Other observations were also consistent with the proposal that the fluency deficit in SA arises from disordered control. First, fluency performance did not correlate with the magnitude of semantic impairment in either task, in contrast to SD; second, many more incorrect responses were produced in SA than SD; and third, the nature of these errors strongly suggest an inability to inhibit incorrect items or control the search/selection process.

## 4 General discussion

Across two semantic syndromes, semantic dementia (SD) and semantic aphasia (SA), we compared and contrasted the effects of typicality, specificity and familiarity on four tasks (picture naming, picture sorting, word–picture matching, fluency) using a new semantic battery (LOFTS). Whilst individual patients within each group were reasonably similar to one another, the comparison across SD and SA revealed a complex pattern of similarities and differences across tasks and patient groups (see Table 3 for a summary). In each case the pattern was consistent with the controlled semantic cognition (CSC) framework articulated in the Introduction. According to this framework, impairments in SD arise from damage to the anterior temporal lobe “semantic hub”, whereas those in SA arise from damage to a fronto-temporoparietal control system that serves to shape the flow of activation in the cortical semantic network. Both forms of damage will produce *representational distortion*: an inability to specify fine-grained patterns of activation in the network, disrupting performance that requires knowledge about the properties that individuate semantic neighbors, especially for low-familiarity items. Because the core impairment in SA lies in the system of control, however, the magnitude of the deficit will scale with tasks or items that require a greater degree of cognitive control—that is, when the task or item requires inhibition of a prepotent response, selection amongst a large number of competitors, or discrimination of a target from very similar competing items. In each task, we have shown how these factors interact to account for the similarities and differences between patient groups. In this sense, we have established at least the face validity of the central hypotheses.

Of course, the CSC framework is not the only approach to understanding the cognitive and neural bases of semantic cognition. In the rest of this Discussion, we consider other hypotheses about the neural bases of semantic abilities and their disorders, and the contributions of anterior temporal versus fronto-temporoparietal systems to these abilities. We briefly review each position and its relation both to the current evidence and the CSC framework. We conclude with a consideration of open questions and future directions.

### 4.1 Semantic aphasia reflects executive dysfunction, a disorder of speech production, or both

One possibility is that semantic aphasia does not constitute a coherent semantic disorder at all, but arises from some mix of executive and speech production disorders. On this view, the visual and verbal comprehension impairments that are a defining feature of the syndrome arise from a general disruption to executive functioning, while anomia and disordered verbal fluency reflect joint contributions of executive and speech production deficits. On its face such an account offers a simple explanation of some of the observed effects, including (1) the coincidence of semantic and executive deficits in SA, (2) the generally poor performance on verbal fluency regardless of specificity or task type, which is to be expected if participants are simply dysfluent, and (3) the frequency and types of errors produced in fluency tasks, which are consistent with disordered control generally.

We suggest at least three general problems with such an account. The first is that it fails to explain other observations in the current study. For instance, it does not explain (1) why specificity does not influence verbal fluency in SA but does influence naming, sorting, and word–picture matching, (2) why familiarity influences specific-level but not basic-level naming or sorting in SA, (3) why typicality exerts qualitatively similar effects in SA and SD in sorting but not naming, or (4) why in SA typical items are disadvantaged in word–picture matching when distractors are proximal, but advantaged when distractors are more distal. These effects all seem to reflect joint effects of executive dysfunction and semantic representational structure, so they are difficult to understand if the impairments arise solely from executive and speech production deficits.

The second problem is that the effects we have documented are observed even in patients who are capable of producing fluent speech. If important aspects of these patterns arise from impaired speech production, we would expect qualitatively different patterns in the fluent versus dysfluent cases. In each task, however, individual patient data adhered well to the group mean performance, and subgroups of this kind were not apparent—fluent and dysfluent cases behaved quite similarly, as also observed in prior work (Jefferies and Lambon Ralph, 2006).

Third, other patient groups with clear executive and verbal production deficits often do not show semantic impairment, suggesting that neither deficit is sufficient to produce SA. For instance, patients with the frontal variant of fronto-temporal dementia are often un- or minimally-impaired on most semantic tasks despite clear deficits on executive tasks (Perry and Hodges, 2000; Rogers et al., 2006). Likewise patients with expressive aphasia, though they often have coincident executive impairments, do not show the nonverbal comprehension impairments that characterize SA (Carthery-Goulart et al., 2012). These observations suggest that SA involves something other than the conjoint disruption of executive function and speech production.

### 4.2 Access versus degraded store

The CSC framework is similar in some respects to the access/degraded storage distinction (Forde and Humphreys, 1995; Gotts and Plaut, 2002; Mirman and Britt, 2014; Warrington and McCarthy, 1983). On this view, semantic impairments can arise either because information in a long-term semantic knowledge store is degraded or because the processes that govern retrieval from such a database are disordered. The different mechanisms align roughly with the distinction we have proposed between damage to the semantic hub versus semantic control systems. When hub neurons and their synapses are damaged, the information they contain is permanently lost and, with sufficient damage, knowledge about associations among sets of attributes is unavailable (not just hard to access). In this sense, patients with SD are similar to classical “storage” patients; indeed, the original storage patients studied by Warrington (1975) likely had semantic dementia. When control systems degrade, the neurons and synapses that encode knowledge of semantic structure and relationships remain intact—the knowledge remains “in the system” but cannot always be properly exploited in a given situation due to noisy and disordered control. In this sense, patients with SA are similar to classical “access” patients. Moreover, some of the phenomena in SA are similar to those observed in access disorders, including (a) low itemwise consistency across and even within tasks, (b) significant susceptibility to cueing, and (c) comorbidity with executive dysfunction.

Is the CSC framework just an access/storage account by a different name? There are at least two reasons for distinguishing the accounts. First, the original storage/access account was developed in a period when the mind-as-computer was a dominant motivating factor in cognitive psychology. The account thus formed itself around certain properties of digital serial computers, including the bright line drawn between storage and retrieval. This distinction is blurred in network-based approaches to memory and to cognition more generally (see Rogers and McClelland (2014), for discussion), so it becomes more difficult to know what is specifically intended by “storage” versus “retrieval” deficits in this context. In addition, while the access/storage distinction concisely summarizes patients' performance characteristics, it does not illuminate the neurocognitive mechanisms that underpin these and other aspects of semantic cognition.

Second, the CSC framework explicitly connects disordered semantic cognition in SA to current theories about cognitive control generally and, in doing so, offers tangible hypotheses about the key computational underpinnings of semantic cognition more generally. A central contribution of the current work is the proposal that semantic impairments in SA scale with the same factors that govern recruitment of cognitive control. Where the task involves generating a highly prepotent response with little competition, semantic impairments will be minimal, even though such a task still requires retrieval from the “semantic store”. The access/degraded store account makes no reference to control demands, and treats retrieval as a process that operates in the same way regardless of the control demands. The CSC framework thus provides a somewhat different perspective on the function and operation of the fronto-temporoparietal systems that are damaged in SA. We view the framework as addressing many of the same issues highlighted within the access/storage tradition, but with reference to neuro-cognitive processing mechanisms similar to those that operate in more contemporary accounts of cognitive control and semantic memory.

### 4.3 Loss of concepts versus loss of associates

A third recent proposal is that, whereas anterior temporal regions (damaged in SD) encode conceptual representations of word/object meanings, inferoparietal regions (sometimes damaged in SA) encode information about thematic relationships amongst familiar items—for instance, knowledge about the relationships between dogs and bones, or between soup and spoons. This view is consistent with a recent large lesion–symptom correlation study which found that generation of associative paraphasias correlated with infero-parietal damage, while generation of coordinate semantic or level errors correlated with superior anterior temporal damage (Schwartz et al., 2011). It is also consistent with some observations from SA in the current and previous studies: whereas associative errors in naming are virtually nonexistent in SD they are not uncommon in SA (Jefferies and Lambon Ralph, 2006), and the “semantic drift” phenomenon observed in verbal fluency may arise when such patients are “lured” by associates of a previously-produced response.

We find the link between inferoparietal lesions and associative errors to be compelling and worthy of further study. We also view these data as potentially consistent with the current proposal. Disordered control involves difficulty in inhibiting competitors that spring to mind, and depending upon the task, such competitors may include associates of a stimulus as well as its semantic neighbors. The increased production of associates for naming and fluency tasks in SA might therefore reflect loss of semantic control. In contrast, the lack of associative errors in SD would result from conceptual degradation, which makes it hard for patients to generate item-specific features or associates. In this situation, generation of associative paraphasias is very unlikely.

The hypothesis that inferoparietal regions are specifically dedicated to knowledge of associative/thematic relationships is more difficult to reconcile with a range of neuropsychological and neuroimaging data, including the current results. First, both SD and SA patients are impaired on tasks that directly probe associative semantic knowledge (Jefferies and Lambon Ralph, 2006) and, indeed, when the two types of information have been directly compared on the same items, SD patients (who have no parietal atrophy) tend to be worse on associative than categorical relationships (Hoffman et al., 2013). Second, a recent fMRI study designed to contrast these two forms of semantic information found that the same semantic network was equivalently activated and argued that these and other types of semantic knowledge may be coded within a single hub-and-spoke framework (Jackson et al., in press). Third, although SA patients produce associative errors in naming, the predominant errors of commission were semantic and level errors. Fourth, the SA group was seriously impaired especially in more specific conditions of the sorting and word–picture matching tasks, in which good performance relies on knowledge of conceptual structure. It is difficult to see how this overall impairment, as well as the observed sensitivity to typicality, familiarity, and specificity, might arise solely from loss of associative knowledge. Finally, some patients with SA have lesions restricted to the frontal lobes, completely sparing infero-parietal cortex, yet these patients show the same pattern of impaired behavior across tasks (see Jackson et al., in press, for further details).

In summary, this brief overview establishes that the CSC framework is related to but distinct from other proposals about the cognitive and neural processes that support semantic cognition.

### 4.4 Open questions and future directions

We believe that, in considering the role of control processes in semantic task performance, the CSC framework provides a particularly useful way of conceptualizing the different causes of semantic dysfunction in these two patient groups. What then are the framework's limitations?

The chief limitation is that it does not specify, in any mechanistic detail, how systems of control and representation interact. As a consequence it is challenging to understand how the effects of representational distortion and disordered control will jointly operate in anything but a qualitative sense. In this paper we have outlined three factors known to influence recruitment of control generally, and attempted to explain how these are also likely to influence recruitment of control in particular semantic tasks. We believe that this establishes the face validity of the approach, but it is not always clear exactly which control demands come into play for which items in which tasks, whether and how different control factors will summate or interact, and so on.

These limitations could be addressed by the development of a more explicit computational model that would allow for simulation of the different varieties of damage (a non-trivial and challenging enterprise to undertake). Influential models consistent with the CSC framework have been described for the semantic network itself (Rogers and McClelland, 2004; Dilkina et al., 2008) and for control processes writ large (Botvinick and Cohen, 2014). What remains is to assess whether models in this tradition can help to explain and predict, in more mechanistic detail, differences between patient groups of the kind we have documented here.

## Figures and Tables

**Table 1:** Background neuropsychological data for the semantic dementia patients.

Case	Age	Sex	YrsEd		WPM	Naming	PPT-words	PPT-pics
***Max score***	***64***	***64***	***52***	***52***
AN	64	M	9		63	53	48	48
LS	60	M	13		63	43	49	49
MC	58	M	20		63	50	49	49
ATe	65	M	20		58	10	44	47
NS	68	F	9		57	13	41	44
MA	63	M	13		57	13	34	42
SJ	60	F	11		51	11	32	45
EK	59	F	10		46	17	36	35
ATh	60	M	10		46	20	31	30
JCh	58	M	10		46	33	36	40
KH	59	M	9		41	22	37	37
GT	70	F	9		32	11	32	37
JG	68	F	11		29	6	28	38
PD	72	F	9		17	4	26	26
MK	66	F	12		11	2	26	33

YrsEd=years of education. WPM=10 alternative forced-choice word-picture matching. PPT=Pyramids and Palm Trees test.

**Table 2:** Background neuropsychological data for the semantic aphasia patients.

Case	Age	Sex	YrsEd	Neuroimaging summary	BDAE classification	WPM	Naming	PPT-words	PPT-pics
***Max score***				
NY	63	M	10	L frontal–temporal–parietal	Conduction	60	55	42	47
SC	76	M	11	L occipital–temporal (and R frontal–parietal)	Anomic/TSA	59	28	51	50
PG	59	M	13	L frontal and capsular (CT)	TSA	58	46	43	42
BB	55	F	11	L frontal and capsular (CT)	MTC	54	10	35	41
KH	73	M	9	L occipital–temporal and frontal	MTC	54	30	39	41
ME	36	F	11	L occipital–temporal	TSA	50	5	39	29
MS	73	F	9	–	Global	46	0	34	41
LS	71	M	10	L temporal–parietal–frontal	TSA	37	5	39	31
KA	74	M	9	L frontal–temporal–parietal (CT)	Global	26	0	44	44
JM	69	F	13	L frontal–temporal–parietal (CT)	TSA	53	30	44	35

YrsEd=years of education. BDAE=Boston Diagnostic Aphasia Examination WPM=10 alternative forced-choice word-picture matching. PPT=Pyramids and Palm Trees test. . TSA=transcortical sensory aphasia. MTC=mixed transcortical.

**Table 3:** Summary of effects across tasks in the SA and SD groups.

*Typicality subset*		*Typicality effect*			
**SA**	**SD**
Picture naming		NULL	+		
Picture sorting	*General*	NULL	+		
*Specific*	++	+		
Word–picture matching	*Distal distractors*	+	+		
*Close distractors*	−	NULL		
			
*Specific*×*Familiarity subset*		*Specificity effect*		*Familiarity effect*	
		**SA**	**SD**	**SA**	**SD**

Picture naming		+	+	+	+
Picture sorting		+	+	NULL	NULL
Word–picture matching		++	+	+	++

+ Denotes the presence of effect; ++ denotes an effect which is stronger in one patient group than that observed in the other.

## Footnotes

Appendix A Supplementary data associated with this article can be found in the online version at doi:10.1016/j.neuropsychologia.2015.04.015.
