# Supplementary material for: Disorders of representation and control in semantic cognition: Effects of familiarity, typicality, and specificity
Source: Neuropsychologia. 2015 Sep;76:220–39. doi: 10.1016/j.neuropsychologia.2015.04.015 (PMC4582808; doi:10.1016/j.neuropsychologia.2015.04.015)
Supplement: Supplementary file 1 — Supplementary material [file mmc1.docx]

**Supplementary materials**

Section 1. Details regarding the development of LOFTS items.

The first step in the formation of the LOFTS was an item-screening process with a group of control participants roughly age- and education-matched to the two patient groups. Reliable assessments that utilize specific concepts are challenging to construct not only because these items are intrinsically the most demanding on the semantic system but also because, as made very apparent by our item screening process, there are considerable individual differences in specific-level knowledge. Depending on each person’s interests, hobbies and profession the level of attained knowledge varies across different semantic categories. Unless one samples from a homogenous group of individuals (patients and controls) with a shared expertise and only probes exemplars from that domain of interest ([cf. Jefferies et al., 2011](#_ENREF_23)), then a different approach is needed. Accordingly, we asked our group of control participants to try to identify a large number of specific-level exemplars (depicted in colour photographs) drawn from many different categories that can be experienced in everyday life, including different types of dog (e.g., labrador, collie, dachshund), birds, cats, water creatures, vegetables, fruits, footwear, types of bread, types of cheese, etc.. Stimuli were only considered for inclusion into the two LOFTS subsets (see below) if at least 70% of the participants were able to name the pictured item at the specific level, unaided (participants were prompted to provide the specific name if they could but were not cued in any other respect). The same participants were also asked to rate the prototypicality of each item with respect to its dominant category (e.g., how typical of a fish is a goldfish) on a 1-7 Likert scale (with 1 being the most typical and 7 the most atypical) and its familiarity (with 1 being the least familiar and 7 the most familiar). Although the most elegant form of such a battery would be to vary all three factors simultaneously, this is difficult, partly because typical exemplars are often referred to using the category name whereas atypical items are labelled using their specific names (e.g., a sparrow will be called “bird” but a penguin will be named “penguin”). Accordingly, the LOFTS battery comprises two subsets of item, one varying typicality and the other varying the familiarity of specific-level concepts.

*LOFTS Typicality subset*: From the large set of screened specific exemplars with reliable recognition, we selected three sets of item that varied rated prototypicality but held familiarity and name agreement constant. Specifically, we selected triplets of items from the same category, comprising a low (rated between 4.5 - 7: e.g., penguin), medium (rated between 3 - 4.5: e.g., pheasant) and high (rated between 1 - 2: e.g., kingfisher) typicality item, which were matched for familiarity and name agreement. This ensured that the same categories of knowledge were sampled equally across the different levels of typicality. The final selection included 16 triplets of items. A list of the items and properties is provided in Appendix A. In the text, we refer to this as the ‘Typicality’ subset, for brevity.

*LOFTS Specific × Familiarity subset*: We selected pairs of specific-level exemplars from the same type of object (e.g., types of cheese), one of which was rated as relatively familiar (5.5 – 7: e.g., cheddar) and the other as being less familiar (1 – 5.5: e.g., brie). The final selection contained 22 such pairs. A list of the items and properties is provided in Appendix B. In the text, we refer to this as the ‘Specific’ subset, for brevity.

All testing materials are available from the first author upon email request.

Section 2. Tables of coefficients from logistic mixed effects models.

In all studies logistic mixed effects models were fit predicting the probability of a correct response on each item, treating patient participant and item as random effects. Magnitude of the semantic impairment was estimated with the Cambrdige 64-item word-picture matching task for non-productive tasks, and with the average accuracy for the naming and word-picture matching components of that battery for tasks involving verbal production. This factor was included as a fixed effect in all models, along with participant group and the factors of particular interest in each task. Important elements of the results and their interpretation are reported in the main text; we here include tables of estimated coefficients for all models and tests of their significance against the null hypothesis.

*Table S1: Coefficients for mixed effects model examining the typicality subset of the picture naming task*

| **Naming typicality subset** |  | B | Serr | Z | p |  |
| --- | --- | --- | --- | --- | --- | --- |
| *Simple effects* | SA (Intercept) | -6.36 | 1.50 | -4.24 | 0.00 | ** |
|  | *WPM* | 0.11 | 0.02 | 4.95 | 0.00 | ** |
|  | *Typicality* | -0.14 | 0.33 | -0.44 | 0.66 |  |
|  | SD | 0.44 | 0.73 | 0.61 | 0.54 |  |
|  |  |  |  |  |  |  |
| *Two-way interaction* | SD : Typicality | -0.51 | 0.26 | -1.95 | 0.05 | ~ |
| NOTE: Coefficients for logistic mixed effects model. In this and subsequent tables, simple effects indicate the additive effects of continuous and discrete predictors, while interaction terms indicate how the effect of these predictors are adjusted for different subconditions. For instance, SD: Typicality indicates how the effect of typicality is adjusted for patients in the SD cohort. SA = semantic aphaisa, SD = semantic dementia, WPM = word-picture matching score. Continuous predictors are italicized. | | | | | | |

*Table S2: Coefficients for mixed effects model examining the specific subset of the picture naming task*

| **Naming specific subset** |  | B | Serr | Z | p |  |
| --- | --- | --- | --- | --- | --- | --- |
| *Simple effects* | SA Hi familiarity (Intercept) | -5.38 | 1.64 | -3.28 | 0.00 | ** |
|  | *WPM* | 0.09 | 0.03 | 3.53 | 0.00 | ** |
|  | Low familiarity | -1.56 | 0.57 | -2.72 | 0.01 | * |
|  | SD | -0.65 | 0.63 | -1.04 | 0.30 |  |
|  |  |  |  |  |  |  |
| *Two-way interaction* | SD : Low familiarity | -0.81 | 0.52 | -1.55 | 0.12 |  |
| NOTE: SA = semantic aphasia, SD = semantic dementia, WPM = word-picture matching score. Italics indicate continuous predictors. | | | | | | |

*Table S3: Coefficients for mixed effects model examining the typicality subset of the sorting task*

| **Sorting typicality subset** |  | B | Serr | Z | p |  |
| --- | --- | --- | --- | --- | --- | --- |
| *Simple effects* | SA General level (Intercept) | 2.62 | 0.88 | 2.99 | 0.00 | ** |
|  | *WPM* | 0.04 | 0.01 | 5.92 | 0.00 | ** |
|  | Specific level | -0.31 | 0.82 | -0.38 | 0.71 |  |
|  | *Typicality* | -0.40 | 0.35 | -1.12 | 0.26 |  |
|  | SD | 3.85 | 1.47 | 2.62 | 0.01 | * |
|  |  |  |  |  |  |  |
| *Two-way interactions* | Specific: *Typicality* | -0.78 | 0.34 | -2.27 | 0.02 | * |
|  | SD: Specific | -3.54 | 1.56 | -2.27 | 0.02 | * |
|  | SD: *Typicality* | -1.19 | 0.55 | -2.18 | 0.03 | * |
|  |  |  |  |  |  |  |
| *Three-way interaction* | SD: Specific: *Typicality* | 1.20 | 0.59 | 2.03 | 0.04 | * |
| NOTE: SA = semantic aphasia, SD = semantic dementia, WPM = word-picture matching score. Italics indicate continuous predictors. | | | | | | |

*Table S4: Coefficients for mixed effects model examining the specific subset of the sorting task*

| **Sorting specific subset** |  | B | Serr | Z | p |  |
| --- | --- | --- | --- | --- | --- | --- |
| *Simple effects* | SA General HF (Intercept) | 0.74 | 0.63 | 1.18 | 0.24 |  |
|  | *WPM* | 0.03 | 0.01 | 2.90 | 0.00 | ** |
|  | Specific level | -0.68 | 0.31 | -2.20 | 0.03 | * |
|  | Low familiarity | 0.30 | 0.47 | 0.64 | 0.52 |  |
|  | SD | 1.55 | 0.46 | 3.39 | 0.00 | ** |
|  |  |  |  |  |  |  |
| *Two-way interactions* | Specific: Low familiarity | 0.00 | 0.46 | 0.00 | 1.00 |  |
|  | SD: Specific | -0.41 | 0.46 | -0.91 | 0.36 |  |
|  | SD: Low familiarity | -0.72 | 0.50 | -1.42 | 0.16 |  |
|  |  |  |  |  |  |  |
| *Three-way interaction* | SD: Specific: LF | 0.13 | 0.63 | 0.21 | 0.84 |  |
| NOTE: SA = semantic aphasia, SD = semantic dementia, WPM = word-picture matching score. Italics indicate continuous predictors. | | | | | |  |

*Table S5: Coefficients for mixed effects model examining the typicality subset of the word-picture matching task*

| **WPM typicality subset** |  | B | Serr | Z | p |  |
| --- | --- | --- | --- | --- | --- | --- |
| *Simple effects* | SA Close (Intercept) | -2.34 | 0.81 | -2.89 | 0.00 | ** |
|  | *WPM* | 0.06 | 0.01 | 5.35 | 0.00 | ** |
|  | Distal | 2.26 | 0.54 | 4.22 | 0.00 | ** |
|  | *Typicality* | 0.31 | 0.24 | 1.28 | 0.20 |  |
|  | SD | -0.53 | 0.50 | -1.06 | 0.29 |  |
|  |  |  |  |  |  |  |
| *Two-way interactions* | Distal: *Typicality* | -0.53 | 0.24 | -2.17 | 0.03 | * |
|  | SD: Distal | -0.58 | 0.65 | -0.89 | 0.37 |  |
|  | SD: *Typicality* | -0.26 | 0.19 | -1.35 | 0.18 |  |
|  |  |  |  |  |  |  |
| *Three-way interaction* | SD: Distal: *Typicality* | 0.07 | 0.30 | 0.23 | 0.82 |  |
| NOTE: SA = semantic aphasia, SD = semantic dementia, WPM = word-picture matching score. Italics indicate continuous predictors. | | | | | | |

*Table S6: Coefficients for mixed effects model examining the specific subset of the word-picture matching task*

| **WPM specific subset** |  | B | Serr | Z | p |  |
| --- | --- | --- | --- | --- | --- | --- |
| *Simple effects* | SA Hi familiarity (Intercept) | -2.23 | 0.70 | -3.18 | 0.00 | ** |
|  | *WPM* | 0.06 | 0.01 | 4.81 | 0.00 | ** |
|  | *Distractor distance* | 0.59 | 0.09 | 6.80 | 0.00 | ** |
|  | Low familiarity | -0.73 | 0.34 | -2.17 | 0.03 | * |
|  | SD | -0.11 | 0.37 | -0.31 | 0.76 |  |
|  |  |  |  |  |  |  |
| *Two-way interactions* | Low familiarity: *Distance* | 0.16 | 0.12 | 1.36 | 0.17 |  |
|  | SD: *Distance* | -0.41 | 0.11 | -3.71 | 0.00 | ** |
|  | SD: Low familiarity | -0.63 | 0.26 | -2.44 | 0.01 | * |
|  |  |  |  |  |  |  |
| *Three-way interaction* | SD: Low fam: *Distance* | 0.03 | 0.15 | 0.20 | 0.84 |  |
| NOTE: SA = semantic aphasia, SD = semantic dementia, WPM = word-picture matching score. Italics indicate continuous predictors. | | | | | | |

*Table S7: Coefficients for mixed effects models examining verbal fluency*

| **General and specific category fluency** | | B | Serr | t |  |
| --- | --- | --- | --- | --- | --- |
| *Simple effects* | SA General (Intercept) | -3.84 | 0.52 | -7.45 |  |
|  | *Composite semantic score* | 0.00 | 0.01 | -0.17 |  |
|  | Specific | 0.28 | 0.24 | 1.15 |  |
|  | SD | -1.00 | 0.60 | -1.66 |  |
|  |  |  |  |  |  |
| *Two-way interactions* | SD: *Composite semantic* | 0.05 | 0.01 | 3.47 |  |
|  | SD: Specific | -0.79 | 0.31 | -2.58 |  |
| **Category and letter fluency** | | B | Serr | t |  |
| *Simple effects* | SA Category (Intercept) | -4.81 | 0.80 | -6.01 |  |
|  | *Composite semantic score* | 0.01 | 0.02 | 0.62 |  |
|  | Letter | 2.46 | 0.46 | 5.34 |  |
|  | SD | -0.82 | 0.93 | -0.89 |  |
|  |  |  |  |  |  |
| *Two-way interactions* | Letter: *Composite semantic* | -0.03 | 0.01 | -2.96 |  |
|  | SD: *Composite semantic* | 0.04 | 0.02 | 1.63 |  |
|  | SD: Letter | 0.58 | 0.28 | 2.10 |  |
| NOTE: Table reports two separate mixed effects models, and includes only those terms whose exclusion reliably reduces model fit. SA = semantic aphasia, SD = semantic dementia. Italics indicate continuous predictors | | | | | |

Section 3. Items in the LOST typicality subset

|  |  |  | **High (1-2)** | | |  | **Medium (3-4.4)** | | |  | **Low (4.5-7)** | | |
| --- | --- | --- | --- | --- | --- | --- | --- | --- | --- | --- | --- | --- | --- |
|  | *Category* |  | Concept | Familiarity | **Typicality** |  | Concept | Familiarity | **Typicality** |  | Concept | Familiarity | **Typicality** |
| 1 | *Animal* |  | tiger | 6.4 | **2.6** |  | squirrel | 6.7 | **3.3** |  | snail | 6.7 | **5.8** |
| 2 | *Animal* |  | mouse | 6.2 | **2.2** |  | chimp | 6.0 | **4.0** |  | hedgehog | 6.4 | **4.5** |
| 3 | *Animal* |  | donkey | 5.6 | **2.0** |  | gorilla | 6.0 | **4.3** |  | tortoise | 5.9 | **5.7** |
| 4 | *Animal* |  | lion | 5.4 | **2.1** |  | elephant | 5.5 | **4.0** |  | frog | 5.7 | **5.7** |
| 5 | *Animal* |  | cheetah | 5.1 | **2.8** |  | hippopotamus | 5.3 | **3.6** |  | kangaroo | 5.3 | **4.9** |
| 6 | *Animal* |  | deer | 5.1 | **1.5** |  | camel | 5.1 | **3.8** |  | bat | 5.1 | **6.1** |
| 7 | *Animal* |  | leopard | 4.7 | **2.1** |  | badger | 4.5 | **3.3** |  | crocodile | 4.8 | **6.3** |
| 8 | *Bird* |  | robin | 6.5 | **1.4** |  | duck | 6.4 | **3.5** |  | swan | 6.5 | **4.6** |
| 9 | *Bird* |  | kingfisher | 5.2 | **2.8** |  | pheasant | 5.6 | **3.6** |  | penguin | 5.2 | **6.4** |
| 10 | *Bird* |  | magpie | 5.1 | **2.4** |  | woodpecker | 4.6 | **3.2** |  | ostrich | 4.8 | **6.1** |
| 11 | *Water creatures* |  | goldfish | 6.0 | **1.8** |  | shark | 6.2 | **3.3** |  | crab | 6.1 | **5.3** |
| 12 | *Water creatures* |  | trout | 4.9 | **1.2** |  | ray | 4.2 | **3.9** |  | seahorse | 4.4 | **6.4** |
| 13 | *Boat* |  | canoe | 5.3 | **2.6** |  | oil tanker (sea) | 4.9 | **3.1** |  | hovercraft | 5.3 | **5.2** |
| 14 | *Boat* |  | rowing boat | 5.4 | **1.9** |  | aircraft carrier | 4.7 | **4.2** |  | submarine | 4.6 | **4.7** |
| 15 | *Land vehicle* |  | lorry | 6.8 | **2.1** |  | tractor | 6.2 | **3.5** |  | caravan | 6.6 | **4.9** |
| 16 | *Aircraft* |  | aeroplane | 6.4 | **1.0** |  | helicopter | 6.4 | **4.3** |  | parachute | 5.7 | **6.1** |
|  |  |  |  |  |  |  |  |  |  |  |  |  |  |
|  | Mean |  |  | 5.63 | **2.03** |  |  | 5.52 | **3.68** |  |  | 5.57 | **5.54** |
|  | SD |  |  | 0.65 | 0.55 |  |  | 0.77 | 0.40 |  |  | 0.75 | 0.67 |
|  | Min |  |  | 6.82 | 2.80 |  |  | 6.70 | 4.30 |  |  | 6.73 | 6.40 |
|  | Max |  |  | 4.73 | 1.00 |  |  | 4.20 | 3.10 |  |  | 4.36 | 4.50 |

Section 3. Items in the LOST specific × familiarity subset

|  | Item | Name Agreement | Familiarity |  | Item | Name Agreement | Familiarity |
| --- | --- | --- | --- | --- | --- | --- | --- |
| 1 | labrador | 1.00 | 6.5 |  | pekinese | 0.80 | 5.4 |
| 2 | robin | 1.00 | 6.5 |  | kingfisher | 0.90 | 5.2 |
| 3 | lion | 0.90 | 6.4 |  | cheetah | 0.90 | 5.1 |
| 4 | tiger | 0.90 | 6.4 |  | panther | 0.80 | 4.6 |
| 5 | shark | 0.90 | 6.2 |  | ray | 0.70 | 4.2 |
| 6 | lobster | 1.00 | 5.8 |  | octopus | 1.00 | 4.6 |
| 7 | crab | 1.00 | 6.1 |  | jellyfish | 1.00 | 4.1 |
| 8 | VW Beetle | 1.00 | 5.8 |  | Rolls Royce | 0.90 | 4.8 |
| 9 | Morris Minor | 1.00 | 5.8 |  | BMW | 0.80 | 4.2 |
| 10 | wellies | 1.00 | 6.7 |  | clogs | 0.90 | 5.2 |
| 11 | spade | 1.00 | 6.3 |  | shears | 0.90 | 4.2 |
| 12 | fork | 0.80 | 6.1 |  | trowel | 1.00 | 4.4 |
| 13 | spoon | 1.00 | 6.6 |  | garlic press | 0.90 | 4.1 |
| 14 | peeler | 1.00 | 6.3 |  | juicer | 0.90 | 4 |
| 15 | pea | 1.00 | 6.4 |  | aubergine | 0.70 | 3.3 |
| 16 | carrot | 1.00 | 6.5 |  | courgette | 0.90 | 4.9 |
| 17 | apple | 1.00 | 6.7 |  | apricot | 0.70 | 4.6 |
| 18 | Fir | 1.00 | 6 |  | maple | 0.70 | 4.1 |
| 19 | daffodil | 0.90 | 6.4 |  | bluebell | 0.70 | 5 |
| 20 | rose | 1.00 | 6.5 |  | lily | 1.00 | 5.4 |
| 21 | baguette | 0.70 | 6 |  | bagel | 0.90 | 3.8 |
| 22 | cheddar | 0.70 | 5.9 |  | brie | 0.90 | 4.9 |
|  |  |  |  |  |  |  |  |
|  | **Mean** | 0.95 | 6.27 |  |  | 0.86 | 4.55 |
|  | **STDev** | 0.10 | 0.29 |  |  | 0.11 | 0.56 |
|  | **Min** | 0.70 | 5.80 |  |  | 0.70 | 3.30 |
|  | **Max** | 1.00 | 6.70 |  |  | 1.00 | 5.40 |

Section 6. Assessing effects of typicality on within- and between-category similarity.

Our account of typicality effects in both disorders depends upon assumptions about the relationship between an item’s typicality and its proximity to items within and between categories. For instance, we suggest that typical items are more similar to, and hence more confusable with, items within the same category, while atypical items are more similar to and more confusable with items from contrasting categories. Rosch et al. (1975) originally observed that typical items share many properties with other category members, but we are not aware of other work testing our particular assumptions about typicality and similarity within and between categories. We therefore tested the assumption for the domain of animals, using the verbal attribute listing norms of Cree, McRae and Seidenberg (2005).

The norms consist of lists of attributes elicited from a large group of participants for 541 natural language concepts, including 133 animals. The authors also collected a variety of psycholinguistic data and ratings for the same items, including ratings of prototypicality for a range of superordinate categories. To test whether more typical items are also more similar on average to other category members, we computed, for each animal item, its mean cosine similarity to all other items. The first panel of Figure S1 shows the relationship between this mean similarity and rated typicality for each animal word. The two measures were positively correlated: more typical items had higher similarity to other category members.

To assess whether less typical items are more similar to items from other categories, we considered, for each animal item, the cosine similarity of the most similar non-animal item in the dataset. The second panel of Figure S1 shows the relationship between this figure and rated typicality across all animal items. The two measures were negatively correlated: items with lower typicality ratings had more similar nearest neighbors from other categories. These analyses thus establish the face validity of our assumptions about typicality and within- versus between-category similarities.


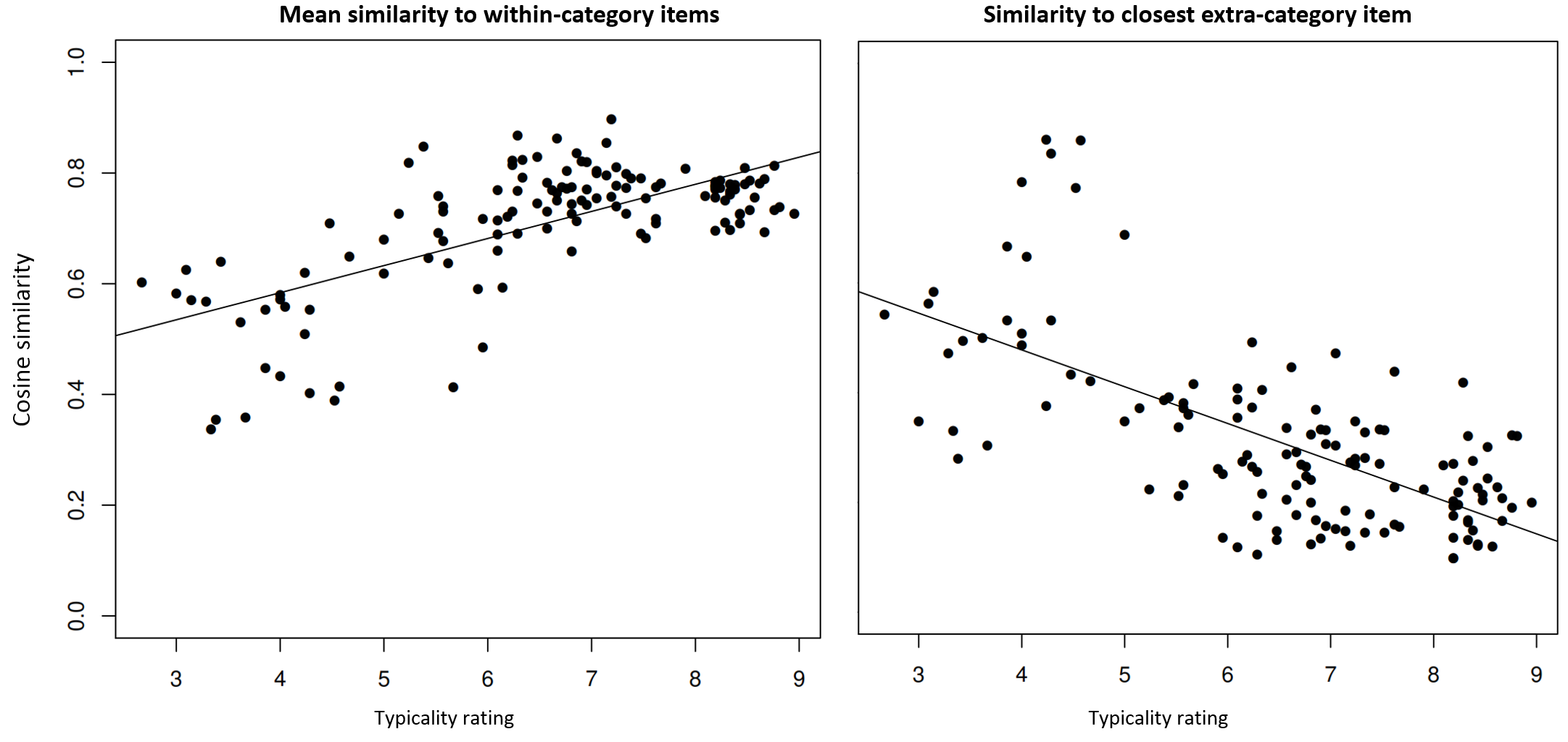


Figure S1. Relationship between rated typicality and cosine similarity to within- and extra-category items. Typicality ratings are mean ratings across participants on a 10-point scale from the Cree, McRae and Seidenberg (2005) dataset.
